# Baduanjin exercise with or without traditional Chinese tuina therapy for nonspecific chronic neck pain: study protocol for a randomised controlled trial

**DOI:** 10.3389/fspor.2026.1787515

**Published:** 2026-03-13

**Authors:** Suhong Zhao, Zhiwei Wu, Ben Cao, Yaping Chang, Yazhou Li, San Zheng, Mengni Shi, Qingguang Zhu, Lingjun Kong, Wuquan Sun, Mitchell Arnold Levine, Min Fang

**Affiliations:** 1 Shuguang Hospital, Shanghai University of Traditional Chinese Medicine, Shanghai, China; 2 Yueyang Hospital of Integrated Traditional Chinese and Western Medicine, Shanghai University of Traditional Chinese Medicine, Shanghai, China; 3 Institute of Tuina, Shanghai Institute of Traditional Chinese Medicine, Shanghai, China; 4 Department of Health Research Methods, Evidence & Impact (HEI), Faculty of Health Sciences, McMaster University, Hamilton, ON, Canada; 5 Department of Medicine, Division of Clinical Pharmacology and Toxicology, Faculty of Health Sciences, McMaster University, Hamilton, ON, Canada

**Keywords:** baduanjin, nonspecific chronic neck pain, randomized controlled trial, study protocol, tuina therapy

## Abstract

**Introduction:**

Non-pharmacological therapies in traditional Chinese medicine (TCM) play an important role in the management of nonspecific chronic neck pain (NCNP). Tuina therapy and Baduanjin exercise, representing passive and active modalities, respectively, have shown benefits in reducing pain, improving cervical mobility, and enhancing quality of life. However, the therapeutic efficacy of Tuina combined with Baduanjin (TCB) approach for NCNP remains insufficiently elucidated. This study aims to determine whether TCB provides superior effects in pain relief and functional outcomes compared with Baduanjin exercise alone.

**Methods and analysis:**

A total of 110 NCNP patients aged 18 to 50 years will be recruited. Participants will be randomly assigned in a 1:1 ratio to either the TCB group or Baduanjin group. Each group will undergo a total of 16 sessions of therapy over 8 weeks, with two sessions per week. Outcomes will be assessed at week 8, 20, and 32 post-randomization. The primary outcome is the pain intensity at week 8, assessed by the visual analogue scale scores (VAS). Secondary outcomes include functional disability (evaluated with the Neck Disability Index), quality of life (assessed using the 12-item Short Form Survey questionnaire), emotional distress (measured by the Hospital Anxiety and Depression Scale), sleep quality (evaluated with the Pittsburgh Sleep Quality Index), work efficiency (assessed using the Work Productivity and Activity Impairment Questionnaire-Specific Health Problem), and safety. The statistical analyses will follow the two-way repeated measures analysis of variance to compare clinical data between groups across different time points.

**Ethics and dissemination:**

The study protocol has received approval from the Ethics Committee of Yueyang Hospital of Integrated Traditional Chinese and Western Medicine, Shanghai University of Traditional Chinese Medicine (2023-143). All study participants will be required to provide written informed consent. The findings of the study will be submitted to a peer-reviewed journal for publication and presented at scientific conferences. Additionally, the participants will receive copies of the results upon requests.

**Clinical Trial Registration**: https://www.chictr.org.cn/, identifier (ChiCTR2300073680).

## 1 Introduction

Nonspecific chronic neck pain (NCNP) is a prevalent condition defined as pain localized to the cervical spine and/or shoulders persisting for more than three months in the absence of identifiable structural abnormalities and is frequently accompanied by motor dysfunction (1). Largely driven by prolonged forward head posture, the global prevalence of NCNP has been rising in recent years, reaching up to 25% in economically developed regions (2, 3). Beyond its high prevalence, NCNP is frequently accompanied by anxiety, depression, and insomnia, leading to diminished quality of life, reduced work productivity, and substantial economic burden (4, 5). In addition to first-line pharmacological agents, such as non-steroidal anti-inflammatory drugs and muscle relaxants (6, 7), various non-pharmacological therapies—including exercise, transcutaneous electrical nerve stimulation, kinesiotherapy, Tuina (traditional Chinese manual therapy), and acupuncture—have been widely applied and demonstrated clinical benefits (8–10).

Tuina involves both soft tissue and spinal manipulations (11). Soft tissue techniques include pressing, pushing, kneading, stroking, friction, and drumming, whereas spinal manipulation is typically characterized by high-velocity, low-amplitude thrusts (12). A systematic review and meta-analysis found that, Tuina significantly reduced neck pain [pooled standard mean difference (SMD) 1.79, 95% confidence intervals (CI), 1.01 to 2.57, *p* < 0.0001] when compared with inactive controls such as waiting list or standard care (13). Baduanjin is a traditional Chinese mind-body exercise that promotes physical and mental health and alleviates illness (14). It consists of a series of structured movements designed to regulate breathing, reduce muscle tension, and enhance joint mobility. Several studies have demonstrated that Baduanjin can alleviate neck pain, improve cervical spine mobility (15–17), and reduce symptoms of anxiety and depression (18). However, controversy regarding efficacy remains due to small sample sizes, methodological limitations, inadequate quality control, low treatment precision, and short follow-up durations (19). Research suggests that combination therapies are often recommended in clinical practice to overcome the limitations and short-term effects of monotherapy (20). Although evidence supports the efficacy of Tuina and physical activity in improving symptoms and psychological outcomes in NCNP, high-quality randomized controlled trials (RCTs) evaluating the combined effects of Tuina and Baduanjin, particularly with long-term follow-up, remain scarce (21, 22).

This RCT aimed to evaluate the efficacy of TCB in reducing pain, disability, and psychological distress in patients with NCNP, with findings expected to clarify whether TCB is superior to Baduanin alone.

## 2 Methods

### 2.1 Study design

This study is a randomized, assessor-blinded, single-center, two-arm, parallel-group superiority trial. The protocol was approved by the Ethics Committee of Yueyang Hospital of Integrated Traditional Chinese and Western Medicine, Shanghai University of Traditional Chinese Medicine (project number: 2023-143), and registered at the Chinese Clinical Trial Registry (ChiCTR2300073680). This study will follow the strict criteria outlined in the Consolidated Standards of Reporting Trials (CONSORT) and the Standard Protocol Items: Recommendations for Interventional Trials (SPIRIT) guidelines.

**Figure 1:**
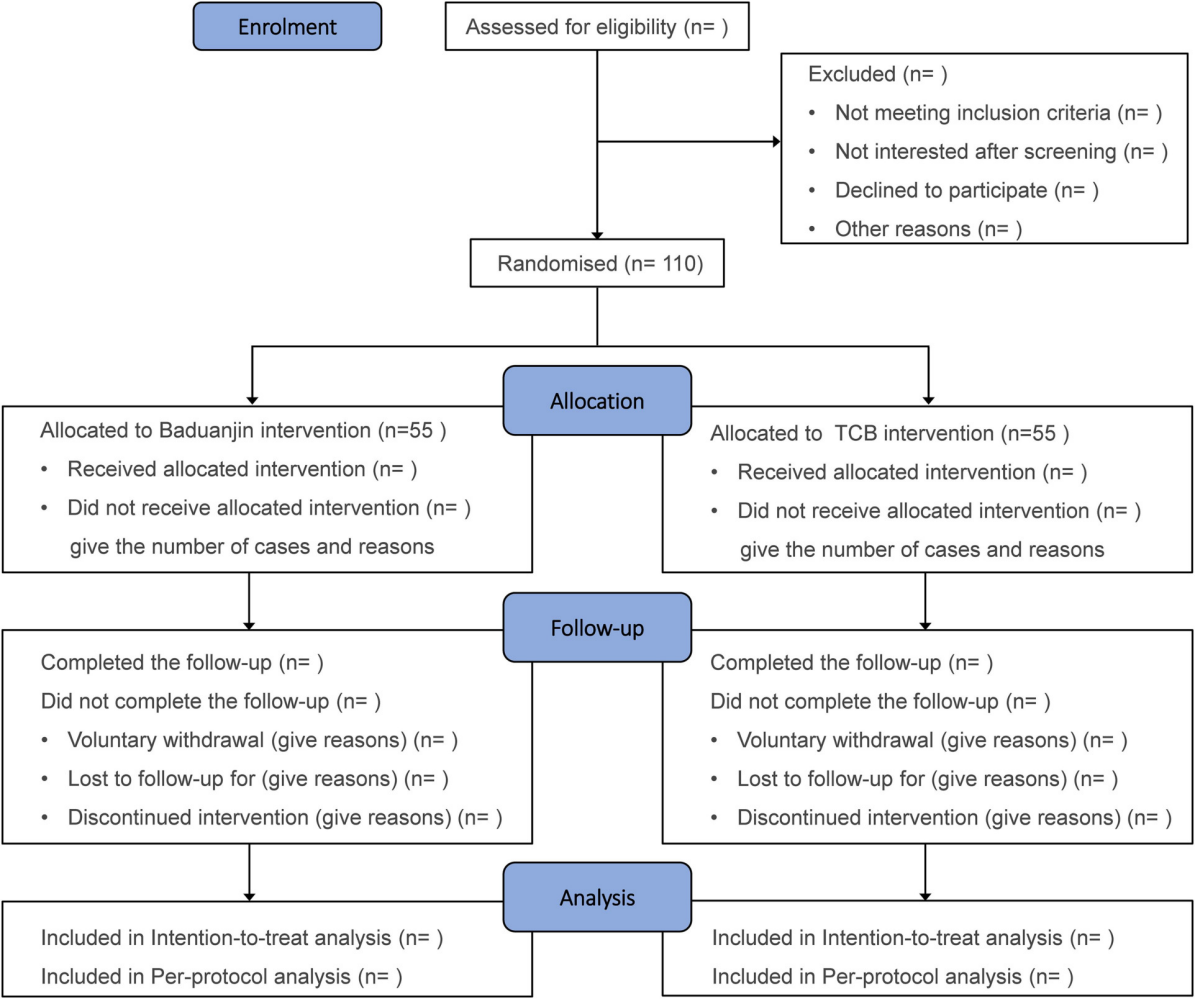
Flowchart of the study. TCB, Tuina combined with Baduanjin.

A total of 110 participants will be recruited from the Tuina Department of Shanghai Yueyang Hospital and randomly assigned (1:1) to either the Baduanjin group or the TCB group. The trial includes an 8-week intervention period and a 24-week follow-up. Baseline characteristics will be collected prior to randomization, and outcomes (pain, functional impairment, quality of life, adverse events) will be assessed at weeks 8, 20, and 32. The study procedures are illustrated in a flowchart (Figure 1), with the intervention and outcome schedule detailed in Table 1.

**Table 1:** Schedule of enrolment, intervention and outcome measures.

	Enrolment and allocation				
	Screening	Allocation	Treatment period	Follow-up period	
Timepoint	Week-1	Week 0	Week 8	Week 20	Week 32
Enrolment					
Eligibility	x				
Demography	x				
Informed consent	x				
Medical history	x				
Physical examination	x				
Allocation		x			
Intervention					
Baduanjin			x		
TCB			x		
Outcomes					
VAS	x		x	x	x
NDI	x		x	x	x
SF-12	x		x	x	x
HADS	x		x	x	x
PSQI	x		x	x	x
WPAI-SHP	x		x	x	x
Adverse events			x		

TCB, Tuina combined with Baduanjin; VAS, Visual Analog Scale; NDI, Neck Disability Index; SF-12, 12-item Short Form Survey; HADS, Hospital Anxiety and Depression Scale; PSQI, Pittsburgh Sleep Quality Index; WPAI-SHP, Work Productivity and Activity Impaimrment Questionnaire: Specific Health Problem.

### 2.2 Participants recruitment

Participants will be recruited through posters in the Tuina Department and advertisements on WeChat, the largest social media platform in China. The poster includes the project title, participant inclusion and exclusion criteria, and contact information, including the recruiter's phone number, WeChat QR code, and address.

The clinical research coordinator (CRC) is responsible for explaining the study protocol and eligibility criteria and conducting a preliminary eligibility assessment for interested individuals. Potentially eligible participants will receive informed consent documents via email, WeChat, or in person, with sufficient time provided for review before making a decision. Eligible participants who confirm interest will attend a baseline visit, where an investigator will review the study protocol, reconfirm eligibility, and obtain written informed consent. To mitigate potential recruitment barriers related to access to Tuina therapy, participants randomized to the Baduanjin group will be assured free Tuina therapy upon completion of the trial. Baseline assessments will include all primary and secondary outcomes along with demographic data, after which participants will be randomized.

### 2.3 Inclusion criteria

Eligible participants who meet the following criteria will be enrolled:

Age between 18 and 50 years old, both male and female.
The primary complaint of pain in the neck region, which starts at the superior nuchal line and continues down to the level of the scapular spine.
NCNP lasting for at least 3 months, with no specific signs of neurological damage or whiplash injury, as defined by the British Medical Journal guidelines.
The average score on the visual analog scale (VAS, scale range, 0–10) over the past week between 3 and 7.
The scores of the neck disability index (NDI, scale range, 0–50) of ≥10.
Voluntarily participate in the trial and sign the informed consent.

### 2.4 Exclusion criteria

Participants who fulfill the subsequent criteria will be excluded:

History of cervical intervertebral disc herniation with associated peripheral nerve symptoms, neck trauma, surgical neck fracture, spinal disease, infectious inflammatory autoimmune diseases, congenital vertebral anomalies; or compression fractures of any location sites.
With abnormal neck appearance, including scars, hyperpigmentation, and papules.
With organic illnesses, such as malignancy, cardiovascular, cerebrovascular, liver, kidney, or genetic disorders.
With mental illnesses, such as anxiety, depression, persecution delusion, and schizophrenia.
Current or past history (within 12 months of screening) of infectious diseases, alcohol, drug, or medication abuse.
Participation in other clinical trials within 3 months prior to enrolment.
Regularly participated in neck pain exercise training over the past 3 months, defined according to the WHO guidelines as more than 150 min of exercise per week (23).
Utilization of any other therapy within 1 month (drug or non-drug).
Women who are pregnant or lactating or planning a pregnancy.
Surgical strategy for the subsequent 8 months.
Not willing to accept or have adverse reactions to Tuina therapy.

The exclusion criteria will apply to individuals who meet one or more of the aforementioned requirements.

### 2.5 Withdrawal, dropout and removal criteria

Participants may withdraw from the trial at any time or be discontinued under the following circumstances, as determined by the clinical Data Monitoring Committee (DMC):

Clinically significant exacerbation of NCNP symptoms that severely impairs daily functioning or safety.
Unanticipated serious adverse events or complications occur during the trial period, including trauma, allergic reactions, or worsening pain.
Occurrence of other serious illness precluding continued participation, such as stroke, hospitalization, surgical interventions, or emergency care.
Unblinding for emergencies.
Withdrawal deemed necessary at the discretion of the researchers.
Participants request to withdraw from the study due to a loss of interest, scheduling conflicts or other personal reasons.

We will strive to contact participants who withdraw for any reason to acquire follow-up data and ensure their safety post-trial.

### 2.6 Randomisation, allocation concealment and blinding

An independent statistician generated the computer-based randomization sequence and prepared sequentially numbered opaque envelopes (SOEs) containing group assignments to ensure allocation concealment. An independent CRC stored and distributed the SOEs, while investigators remained blinded to group allocation. Following informed consent and baseline assessment, a designated researcher will assign each participant a unique study ID and open the corresponding envelope to reveal the allocation (“Baduanjin” or “TCB”). Given the nature of the interventions, intervention implementation personnel and participants cannot be blinded to group allocation. Evaluators, data entry personnel, and statisticians will remain blinded throughout the study to ensure objectivity.

### 2.7 Sample size calculation

The sample size was determined for the primary outcome, mean pain intensity over the past week measured by the 0–10 VAS at 8 weeks post-randomization. We made an assumption of the anticipated between-group mean difference and standard deviation (SD) for the primary outcome (VAS) based on both literature and internal review of electronic patient records. An effect size of −1.2 points (95% CI, −1.6 to −0.8) was reported for tuina therapy combined with Yijinjing exercise (24) (a different exercise than Baduanjin, but also based on traditional Chinese exercise) compared with tuina therapy alone. Previous literature confirmed that for the measurement of bone and joint pain, 1.5 points can represent clinically important difference (MCID) (25). Due to the lack of direct previous research results on Tuina with or without Baduanjin, we set the smallest threshold difference than the results from literature or real-world data suggest. Therefore, a between-group difference of −1.5 in mean change at Week 8 and standard deviation1.1 to calculate the sample size. Calculations were performed using PASS 15 software with reference to Sample Size Calculations in Clinical Research (26). Assuming two groups, a two-sided significance level of 0.05, 80% power, and a 80% follow-up rate, 55 participants per group were required, yielding a total sample size of 110.

### 2.8 Intervention

Interventions will be conducted in dedicated treatment rooms at Yueyang Hospital by therapists who have undergone standardized training. Participants will receive 16 sessions over 8 weeks with 2 sessions per week and 30-minute per session. The intervention period will last 8 weeks. No further study treatment will be provided thereafter. Outcome assessments at weeks 20 and 32 reflect 12 and 24 weeks post-intervention follow-up. Before the intervention, all participants will receive standardized health education on cervical spine posture, including avoiding vigorous acceleration-deceleration activities, using pillows that support physiological curvature, and minimizing prolonged forward head posture, flexion, or tilting. During the intervention, session attendance will be documented in standardized logs, though no additional experimental data will be collected. Participants who miss sessions will be contacted to determine reasons and encourage adherence. While the use of additional therapies is discouraged but not prohibited, any concomitant treatments will be systematically recorded.

#### 2.8.1 Baduanjin group

The Baduanjin intervention protocol was developed based on standardized curricula of traditional Chinese medicine universities and validated regimens from prior studies demonstrating its efficacy in pain populations. The protocol includes five specific postures: “holding the hands high with palms up to regulate the internal organs”, “posing as an archer shooting both left-and right-handed”, “holding one arm aloft to regulate the functions of the spleen and stomach”, “looking backwards to prevent sickness and strain” and “thrusting the fists and making the eyes glare to enhance strength”. The five postures incorporate multi-directional cervical stretching and stability training, targeting key cervical muscle groups. The five selected movements primarily target cervical and shoulder musculature and were chosen to enhance feasibility and adherence while minimizing lower-limb loading. Each session begins with a 5-minute warm-up to reduce the risk of muscle strain, followed by sequential performance of the five postures. To ensure competency and safety, participants will receive standardized Baduanjin instruction from a certified physiotherapists (QZ) through instructional sessions, pamphlets, videos, and individual guidance, then do exercise by themselves. QZ has completed postdoctoral research in human exercise science at the University of Ottawa and the Shanghai Institute of Sport, along with a strong professional background in exercise practical and teaching. Participants will be encouraged to continue regular Baduanjin practice after completing the 8-week program. Detailed intervention protocols are provided in the online Supplementary File 1.

#### 2.8.2 TCB group

The Tuina intervention will be performed by three licensed Tuina physicians from the Tuina department of Yueyang hospital. To ensure treatment standardization, a 12 h technical training program will be conducted over two weeks (three sessions per week), comprising lectures, video demonstrations, and practical exercises. Following training, physicians will undergo an assessment, with only those who pass deemed eligible to deliver the intervention. Training and evaluation will be supervised by Professor MF, editor-in-chief of the Chinese Tuina National Planning Textbook and an expert with extensive clinical and teaching experience.

The Tuina intervention will last 20 min and comprise three sequential steps: local manipulation, acupressure manipulation and spinal manipulation. Specifically, rolling, pressing-kneading, plucking and grasping manipulation will be applied for 11 min to relax the muscles of the suboccipital, cervical, shoulder, and upper back regions. Subsequently, acupressure manipulation will be administered to the acupoints—including Fengchi (GB20), Fengfu (DU16), Dazhui (DU14), Jianjing (GB21), Tianzong (SI11) and Jiaji (EX-B2)—for about 1 min per acupoint. Acupoint localization will follow the WHO Standard Acupuncture Point Locations in the Western Pacific Region (ISBN: 9787117123327). Illustrations of the acupoints are provided in the Supplementary Materials, and further details of the intervention are available in online Supplementary File 2.

### 2.9 Outcomes

The primary efficacy evaluation will focus on the assessment of average pain intensity in NCNP patients over the past week at 8, 20, and 30 weeks after randomization. Secondary outcomes will include quality of life, emotional distress, sleep quality, and work efficiency.

At each assessment point, data will be collected by independent clinical research coordinators who are blinded to group allocation.

#### 2.9.1 Primary outcome

The primary outcome is the self-reported average pain intensity over the past week at week 8 post-randomization, assessed using the VAS. VAS is a 10-cm horizontal scale anchored by [0] on the left end, indicating “no pain”, and [10] on the right end, indicating the “worst pain”. Higher scores reflect greater pain intensity relative to baseline (27). The VAS was demonstrated to be a valid, responsible, concise and easily interpreted outcome measure with intraclass correlation = 0.92 (28).

#### 2.9.2 Secondary outcomes

##### 2.9.2.1 Neck disability index (NDI)

NDI will be used to assess functional limitations associated with neck pain (29, 30). The questionnaire consists of 10 items addressing pain intensity, personal care, lifting, reading, headache, concentration, work ability, sleep quality, driving, and recreational activities. Each item is scored on a 6-point scale from 0 to 5, where 0 indicates no limitation and 5 indicates complete disability (31). The total scores range from 0 to 50, with higher scores indicating greater functional impairment (32). The NDI demonstrates good reliability and validity, making it an important tool for cervical spine disability measurement (33).

##### 2.9.2.2 12-item short form survey questionnaire (SF-12)

Health-related quality of life will be assessed using the SF-12, which comprises 12 items yielding two summary scores: the Physical Component Summary (PCS) and Mental Component Summary (MCS) (34, 35). Each item is scored on a 5-point scale, and scores for physical and mental health are calculated on a 0–100 scale using the standard American scoring method, with higher scores indicating better self-perceived quality of life (36).

##### 2.9.2.3 Hospital anxiety and depression scale (HADS)

HADS was utilized to evaluate NCNP-induced anxiety and depression using 14 items, with 7 assessing anxiety and 7 assessing depression. Each item is scored on a 0–3 scale based on symptom frequency over the past month, with higher scores indicating greater severity of anxiety or depression (37).

##### 2.9.2.4 Pittsburgh sleep quality Index (PSQI)

The PSQI is a widely employed self-report instrument for sleep quality assessment. PSQI comprises 18 items across seven components: subjective sleep quality, sleep latency, sleep duration, sleep efficiency, sleep disturbances, use of sleep medication, and daytime dysfunction (38). Each item is scored from 0 to 3, and the global PSQI score ranging from 0 to 21, with higher scores indicating poorer sleep quality. Scores of 0–5 indicate good sleep quality, 6–10 fair quality, 11–15 poor quality, and 16–21 very poor quality.

##### 2.9.2.5 Work productivity and activity impairment questionnaire-specific health problem (WPAI-SHP)

WPAI-SHP will be used to investigate absenteeism, work attendance, productivity loss, and daily activity impairment due to illness in the seven days prior to the interview. It can be divided into four scores: absenteeism due to illness, work loss due to illness (when attending work), overall work loss, and activity impairment. All scores are multiplied by 100 and expressed as a percentage, with higher values indicating greater impairment and lower work productivity (39, 40).

##### 2.9.2.6 Cervical range of motion (CROM)

Cervical flexion and extension dysfunction is the most common clinical manifestation of nonspecific chronic neck pain. This study will take the cervical range of motion as one of the secondary evaluation indicators. CROM data will include maximum flexion angle and maximum extension angle of cervical. These measurements will be obtained using an advanced infrared high-speed motion capture system (Vicon Motion System, Oxford Metrics, UK), which provides high-precision, instrumented assessment of cervical mobility. Because the operation and maintenance of this technology is costly, it is not feasible to apply it to all participants in the present trial. CROM data will be obtained in approximately 25% of participants in each study group at week 0 and week 8.

### 2.10 Safety assessment

Before each treatment session, two research evaluators will document any discomfort reported by participants during or after the previous session and record it as an adverse event (AE), including increased pain, muscle soreness, or exacerbated fatigue. Changes in medication use or additional interventions will also be documented in the case report form (CRF). Serious AEs (requiring hospitalization, being life-threatening, or resulting in death) will lead to trial termination and immediate reporting to the ethics committee. Adverse events will be graded according to a predefined severity scale (Grade 1–5), adapted from Common Terminology Criteria for Adverse Events (CTCAE) version 5.0. Grade 1 events will be monitored without intervention. Grade 2 events will lead to temporary suspension of treatment and symptomatic management. Grade 3 or above will result in immediate discontinuation and reporting to the ethics committee within 24 h. An independent Data Safety Monitoring Board (DSMB) comprising a clinician, a biostatistician, and an ethics expert will regularly review safety data and may recommend early termination.

### 2.11 Statistical analysis

All statistics were performed by independent statisticians using SPSS software (version 24.0, SPSS Inc., Chicago, IL, USA). The primary and secondary outcomes will be analyzed based on the intention-to-treat principle. Primary outcome analysis will be adjusted for baseline VAS and other prespecified covariates using ANCOVA or multiple linear regression if baseline imbalance is observed. Based on MCID of 1.5 points, the proportion of participants achieving MCID will be calculated. Occupation (sedentary vs. non-sedentary work) will be included as a stratification variable in WPAI-SHP analysis. All between-group comparisons will base on the change score of a specific time point and the baseline. Baseline characteristics will be summarized in a table and compared between groups to assess comparability. Missing values for dropout cases will be imputed using either the last observation carried forward method if the dropout rate is below 5% or the multiple imputation method for a dropout rate of 5% or higher. Descriptive statistics will be presented as mean ± SD for continuous variables that follow a normal distribution and as median (IQR) for those that do not follow a normal distribution, based on the results of normality tests. For ordinal variables, the data will be presented as median (IQR) or frequency (*n*, %) depending on the nature of the data. Categorical variables will be summarised as *n* (%). To analyse trends over time and the interaction between groups and times, two-way repeated measures analysis of variance will be used to compare clinical data at multiple observation time points.

### 2.12 Data collection and management

Raw data will be recorded in CRFs with high accuracy. Baseline variables including age, sex, disease duration, baseline pain intensity, occupational posture load, and prior treatment history will be recorded. Two independent data managers will collect the CRFs within a specified time frame, enter the data into Excel, and verify accuracy. Both will receive comprehensive training in data management and security and will be regularly reviewed by the Data Monitoring Committee (DMC). Electronic data will be stored on a password-protected server, and paper records in locked filing cabinets. To safeguard patient privacy, all data will be de-identified before storage and analysis. An Electronic Data Capture (EDC) system will be used, with all data entries and modifications automatically logged.

### 2.13 Quality control

Quality control measures will be implemented throughout the trial to ensure methodological rigor. All participating research staff will undergo standardized training in assessment methods, research procedures, and monitoring protocols. Only those who complete the training and pass competency assessments will be permitted to participate in the trial. Training and competency assessments will be jointly supervised by a licensed Tuina therapist (≥10 years clinical experience) and a certified Baduanjin instructor (≥10 years of practice and teaching) with a title of intermediate level or above.The Ethics Committee of Yueyang Hospital of Integrated Traditional Chinese and Western Medicine, Shanghai University of Traditional Chinese Medicine, is responsible for the implementation and safety monitoring of this trial.

## 3 Discussion

NCNP is a common condition associated with high global morbidity, substantially impairing quality of life and work productivity (41). Although pharmacological therapy remains the first-line strategy, its long-term use is limited by high recurrence rates, poor sustained efficacy, adverse effects, and economic burden (42). Tuina therapy and Baduanjin exercise, two traditional Chinese medicine based non-pharmacological interventions, have long been applied in both health care and clinical treatment (43). These therapies could alleviate chronic pain, improve quality of life, and reduce economic burden with the added advantages of simplicity, accessibility, and minimal adverse effects (44).

According to traditional Chinese medicine, neck pain is attributed to impaired systemic circulation, and mechanical imbalance of cervical muscles and skeletal structures (19). The Tuina therapy employed in this study include local manipulation, acupressure manipulation and spinal manipulation, aim to restore musculoskeletal balance, relieve pain, and improve mobility (45). when compared with active therapies such as exercise or traction, Tuina did not significantly differ in reducing neck pain (SMD 0.13, 95% CI −0.38 to 0.63, *p* = 0.63) (13). The evidence indicates short-term benefit of Tuina relative to inactive treatment but no clear superiority over active interventions, with conclusions limited by small sample sizes, methodological weaknesses, and short follow-up (13). Baduanjin, one of the most widely practiced traditional Chinese exercises, is believed to enhance systemic circulation of qi and blood, strengthen muscles, and increase cervical range of motion (46). Therefore, this well designed RCT is designed to evaluate whether the combined intervention provides superior benefits and yield significant clinical efficacy compared with exercise alone. Baduanjin was selected as the active comparator because exercise therapy is recommended as first-line non-pharmacological treatment for chronic neck pain. This design allows us to determine whether adding a resource-intensive passive intervention (tuina) provides additional benefit beyond active exercise alone.

The aim of this study was to evaluate the comparative effectiveness of TCB vs. Baduanjin in alleviating pain severity, improving functional outcomes, and monitoring adverse events among patients with NCNP. Validated scales and questionnaires were employed to comprehensively assess clinical outcomes. Given that pain is the predominant symptom of NCNP, the primary outcome was pain intensity, measured using the VAS. In addition to statistical significance, the clinical importance of treatment effects will be interpreted against established MCID thresholds for VAS. Secondary outcomes included functional limitations assessed by the NDI, health-related quality of life evaluated by the SF-12, anxiety and depression measured by the HADS, sleep quality assessed by the PSQI, and work productivity and daily activity impairment measured by the WPAI-SHP.

This study represents the first RCT to evaluate TCB for the management of NCNP. Currently, the combined use of Tuina therapy and Baduanjin exercise has not been recommended in existing neck pain management guidelines, primarily due to a lack of supporting evidence. Identifying a cost-effective intervention with durable benefits could substantially influence clinical practice for NCNP. The findings of this trial are expected to provide high-quality evidence on the efficacy and safety of TCB, thereby supporting its wider clinical application and potentially informing future guideline development for NCNP management.

This research protocol offers several notable strengths. First, the trial will be conducted at No. 1 Tuina medical institution in China with over 200 thousand outpatient visits annually, ensuring robust patient availability. Second, Tuina, as a non-pharmacological therapy, eventhough is generally versatile in application, may pose potential risks and is not suitable for all individuals. Accordingly, this protocol established targeted exclusion criteria, omitting participants with postoperative conditions, skin disorders, or severe organic diseases. Third, both short-term (8-week) and maintenance effects (32-week) outcome assessments are included, allowing evaluation of sustained efficacy. The 32-week assessment represents outcomes 24 weeks post-intervention, reflecting maintenance rather than continuous treatment effects. Fourth, Intervention training and quality control will be overseen by the first Tuina practitioner in China to hold a doctoral degree, ensuring standardized procedures and methodological rigor. Finally, the study adopts a comprehensive assessment framework (physical function, psychological state, and social participation), thereby providing a multidimensional understanding of effects of TCB.

Limitations of this protocol include the absence of blinding and the limited universality of the results. First, due to the inherent characteristics of the interventions, blinding of participants and therapists was not feasible, and may introduce potential issue of differences between groups. Restricting eligibility to participants with moderate pain (VAS 3–7) may limit generalizability to patients with mild or very severe pain. This strategy was chosen to enrich the sample with individuals most likely to benefit and to reduce floor and ceiling effects. Future studies should consider broader inclusion criteria and stratified analyses by baseline pain severity.

## Supplementary material

Supplementary Document 1 SPIRIT Checklist.
