# Supplementary material for: Baduanjin exercise with or without traditional Chinese tuina therapy for nonspecific chronic neck pain: study protocol for a randomised controlled trial
Source: Front Sports Act Living. 2026 Mar 13;8:1787515. doi: 10.3389/fspor.2026.1787515 (PMC13021794; doi:10.3389/fspor.2026.1787515)
Supplement: Supplementary file 1 [file table1.doc]

- **Baduanjin exercise**

Step 1. Holding the hands high with palms up to regulate the internal organs

| **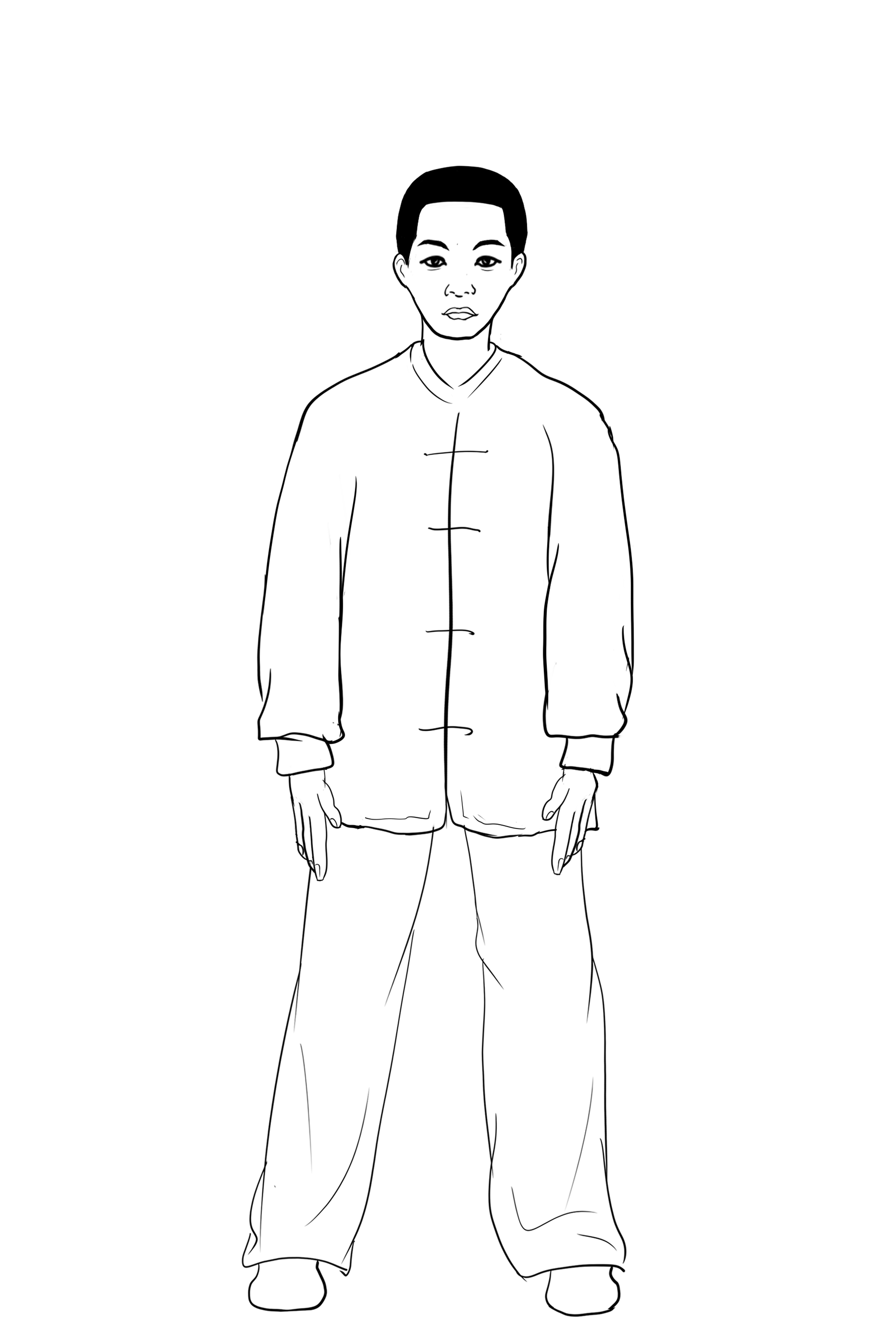**  (a) | (1) Preparatory posture. Stand with the feet positioned approximately shoulder-width apart. Maintain slight flexion of the knees, ensuring that the patellae do not extend anteriorly beyond the vertical line of the toes. Keep the head in a neutral position with the gaze directed forward. Allow breathing to remain natural and unforced, while promoting generalized muscular relaxation and sustained attentional focus. |
| --- | --- |
| **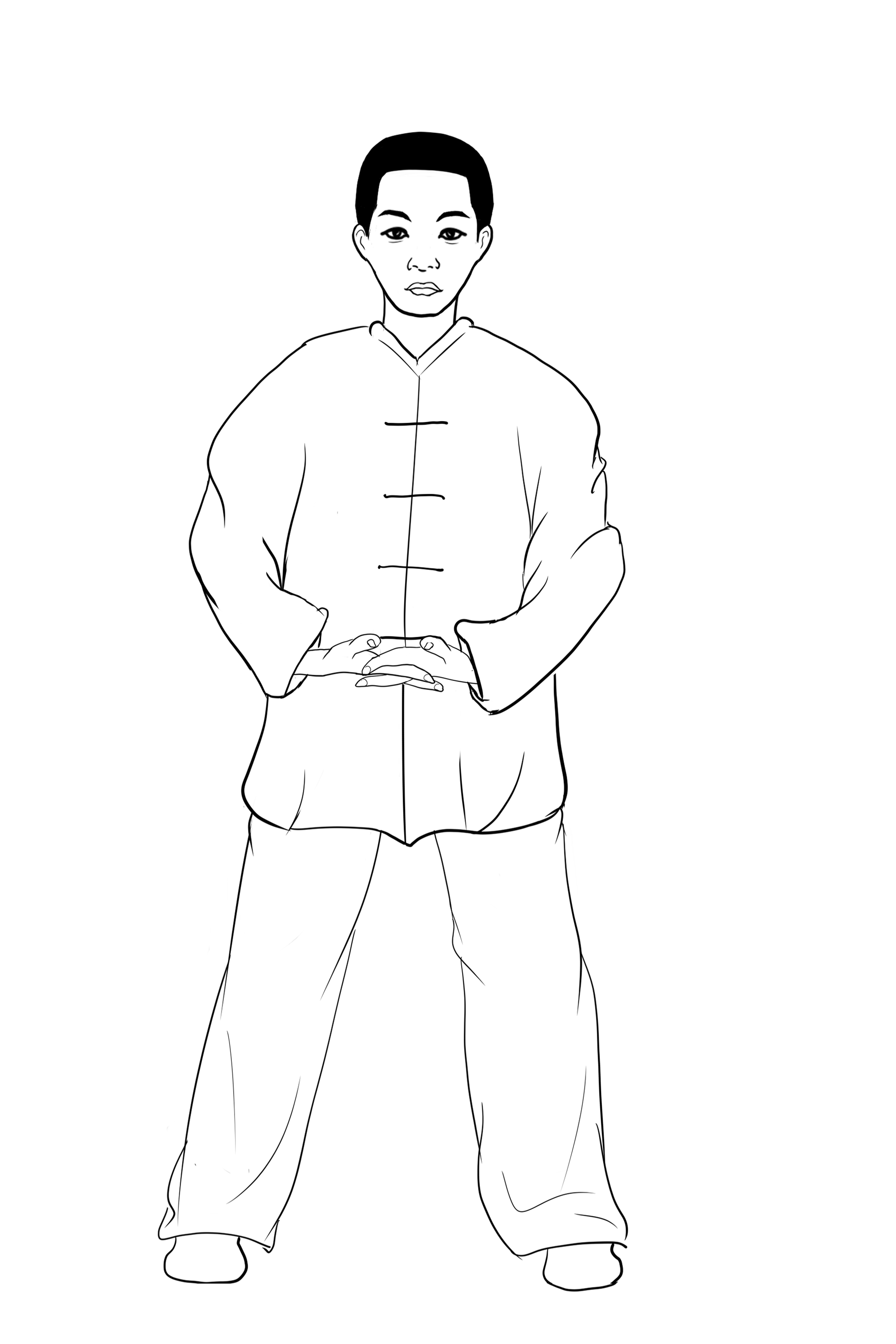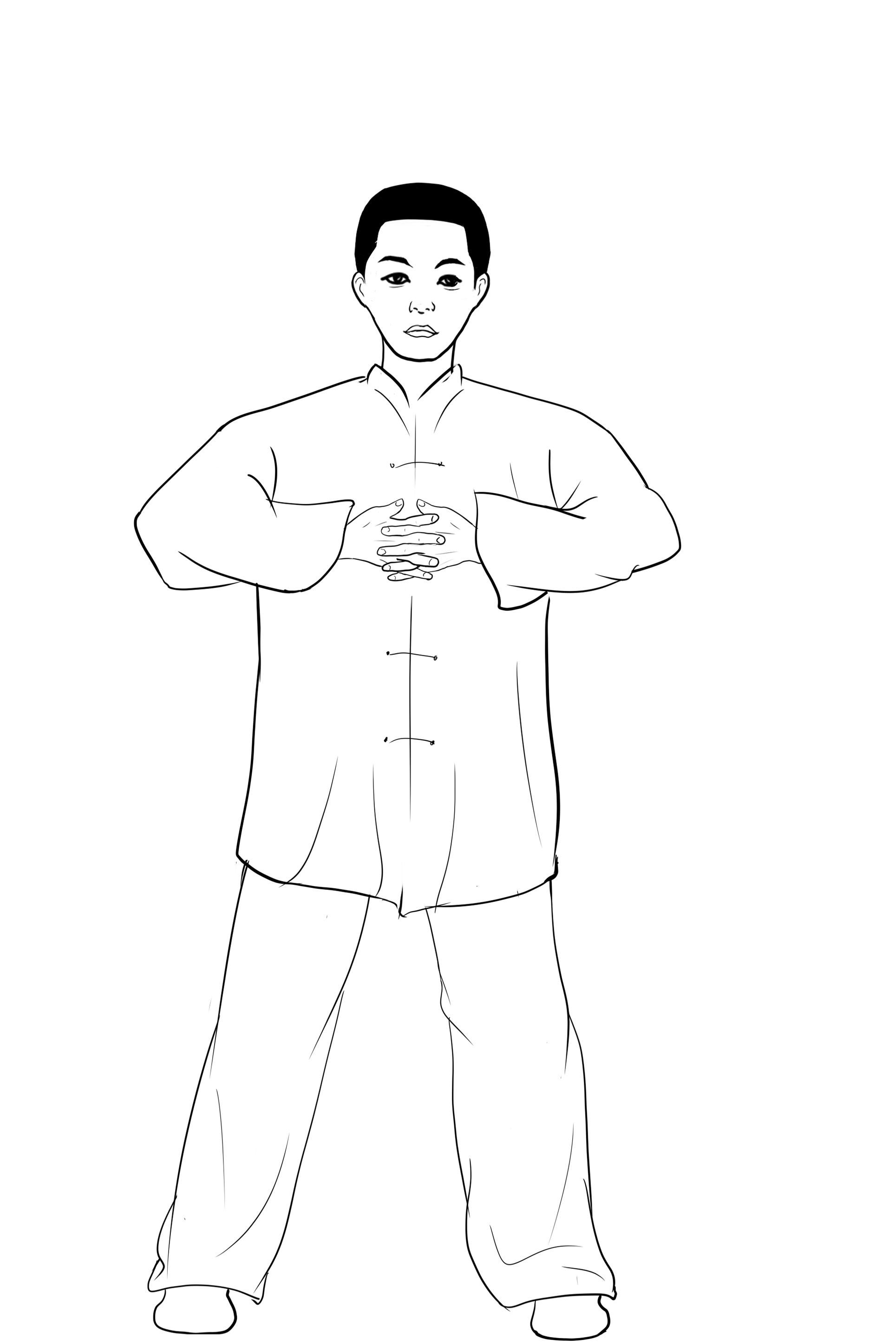**  (b) (c)  **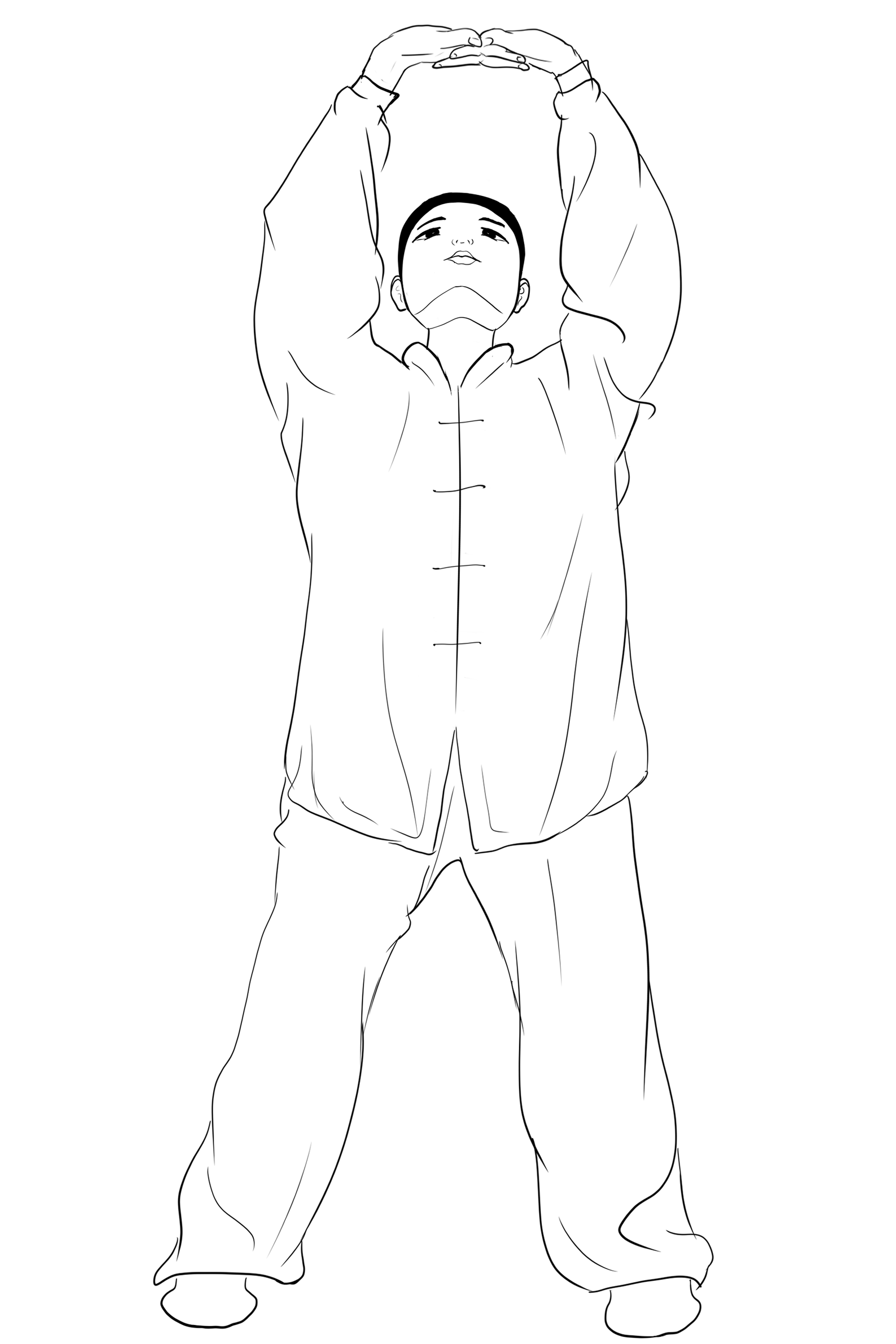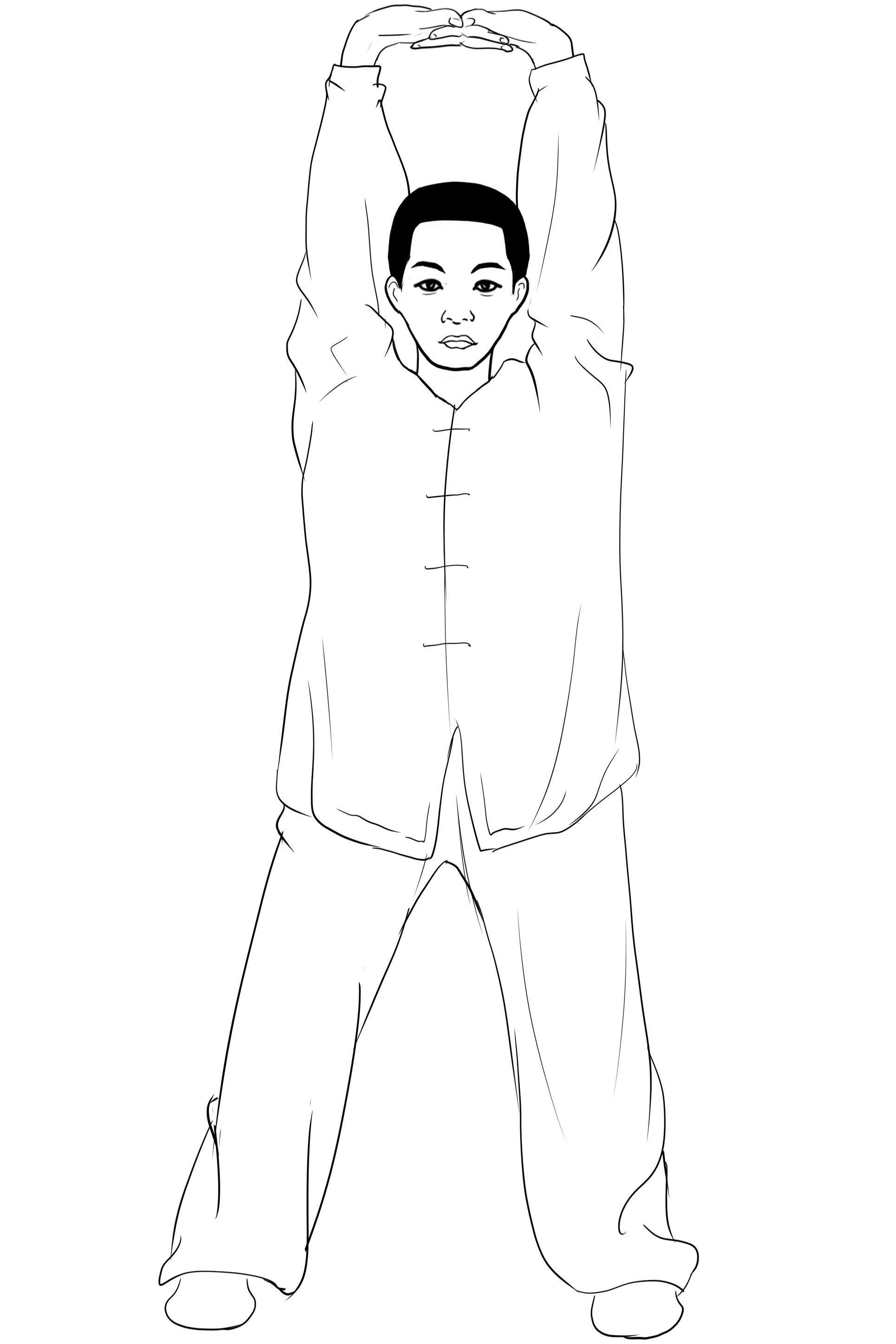**  (d) (e) | (2) Holding the hands high with palms up. Interlace the fingers with the palms oriented upward and slowly raise the hands from the lower abdominal region (b). Upon reaching chest level, rotate the palms inward (c), then continue to rotate them downward, outward, and upward until the arms are fully extended overhead. Concurrently, gradually lift the heels from the ground while gently tilting the head backward, directing the gaze toward the dorsum of the hands (d). Conclude the movement by lowering the heels to the floor and returning the head to a neutral position (e). |
| **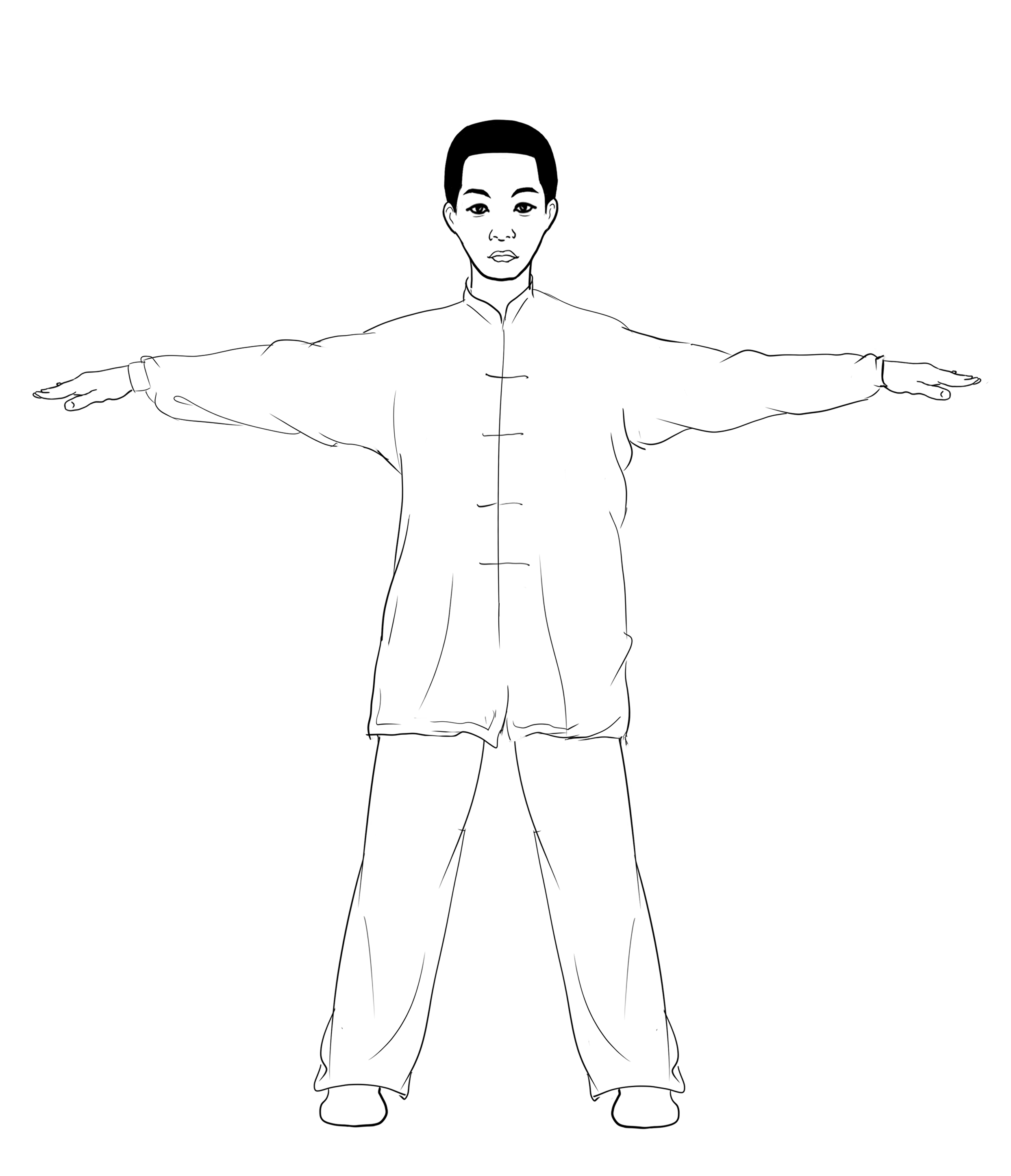**  (f)  **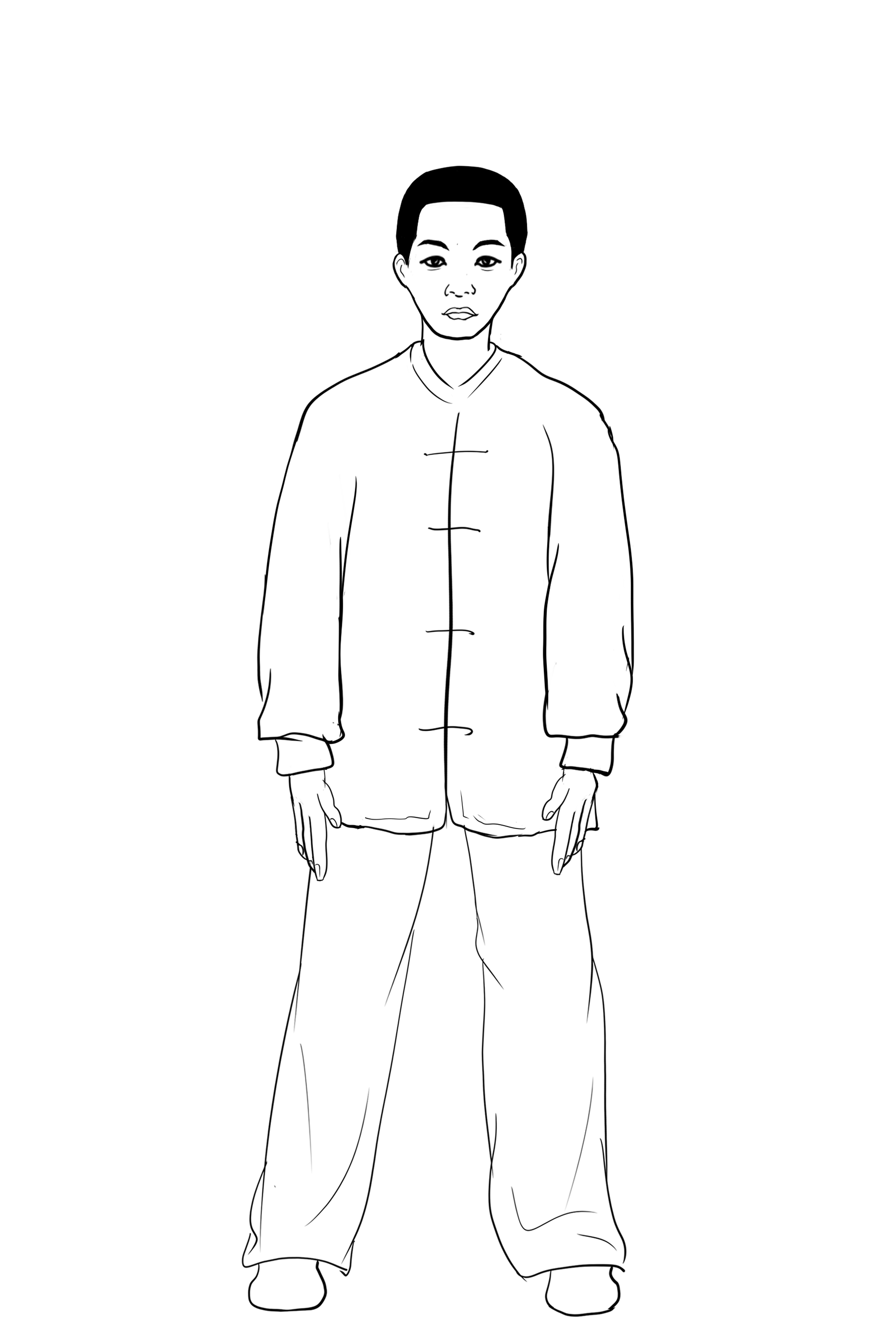**  (g) | (3) Bilateral upper limb return phase. Execute controlled glenouuuhumeral depression with synchronized lowering of upper extremities through shoulder adduction and elbow extension (f). Simultaneously achieve plantar surface recontact through eccentric calcaneal lowering (g). The movement culminates in full return to the preparatory posture (as specified in Step 1). |
| The above actions need to be completed in order and repeated 6 times. | |

Step 2. Posing as an archer shooting both left-and right-handed

| **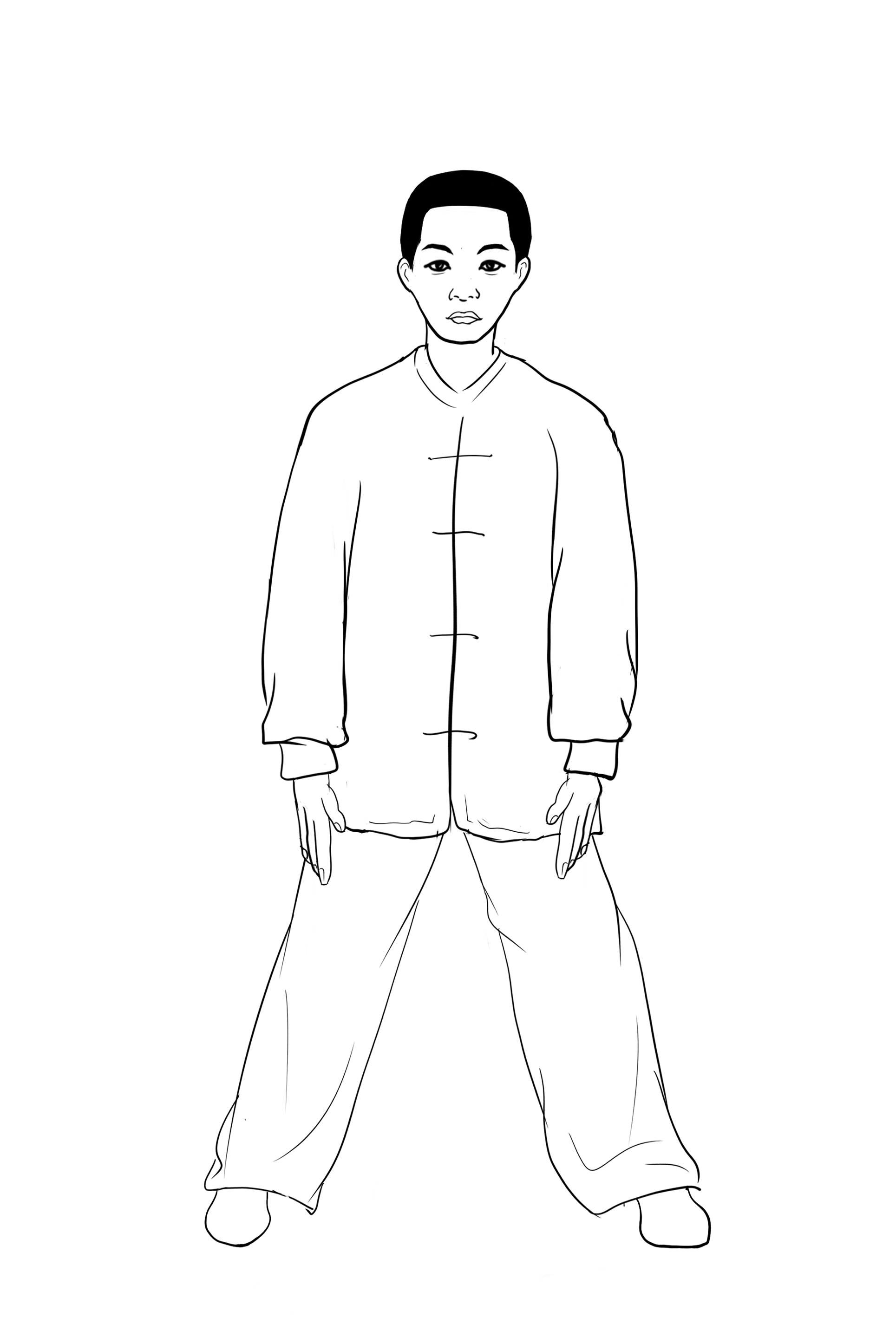**  (a) | (1) Preparatory posture. The same as (1) in Step 1 except that the distance between the two feet is 1.5 times the width of the shoulders. |
| --- | --- |
| **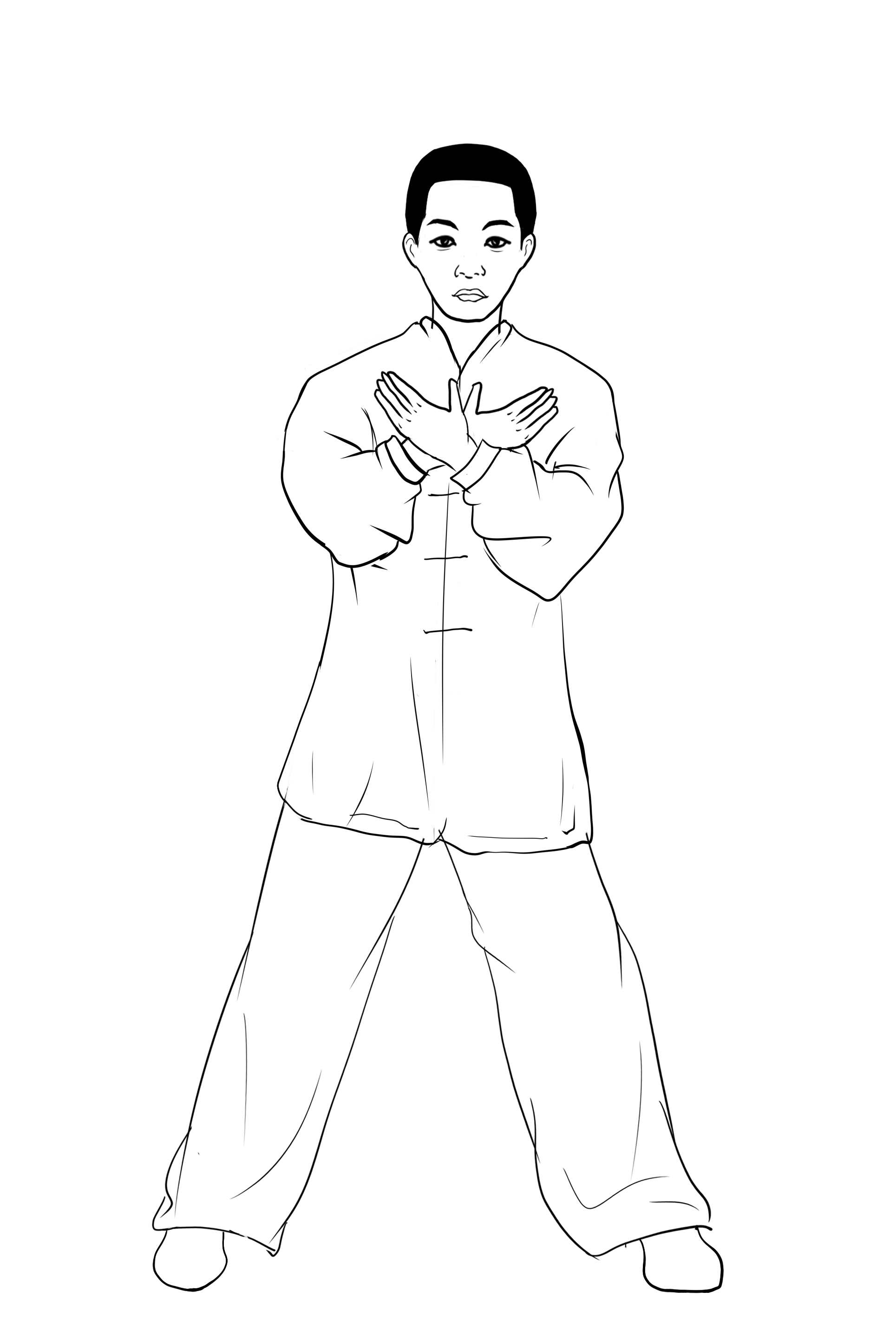 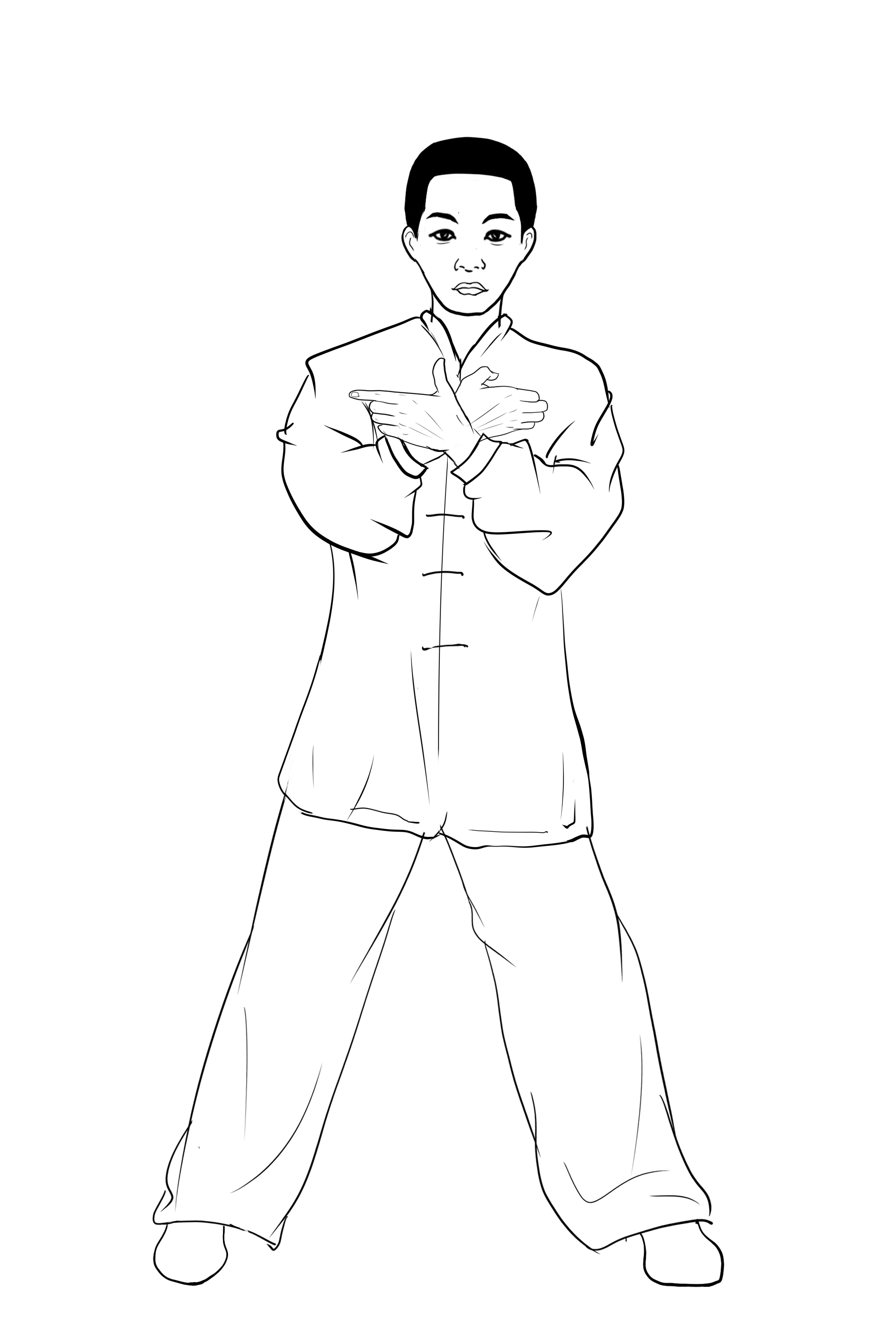**  (b) (c)  **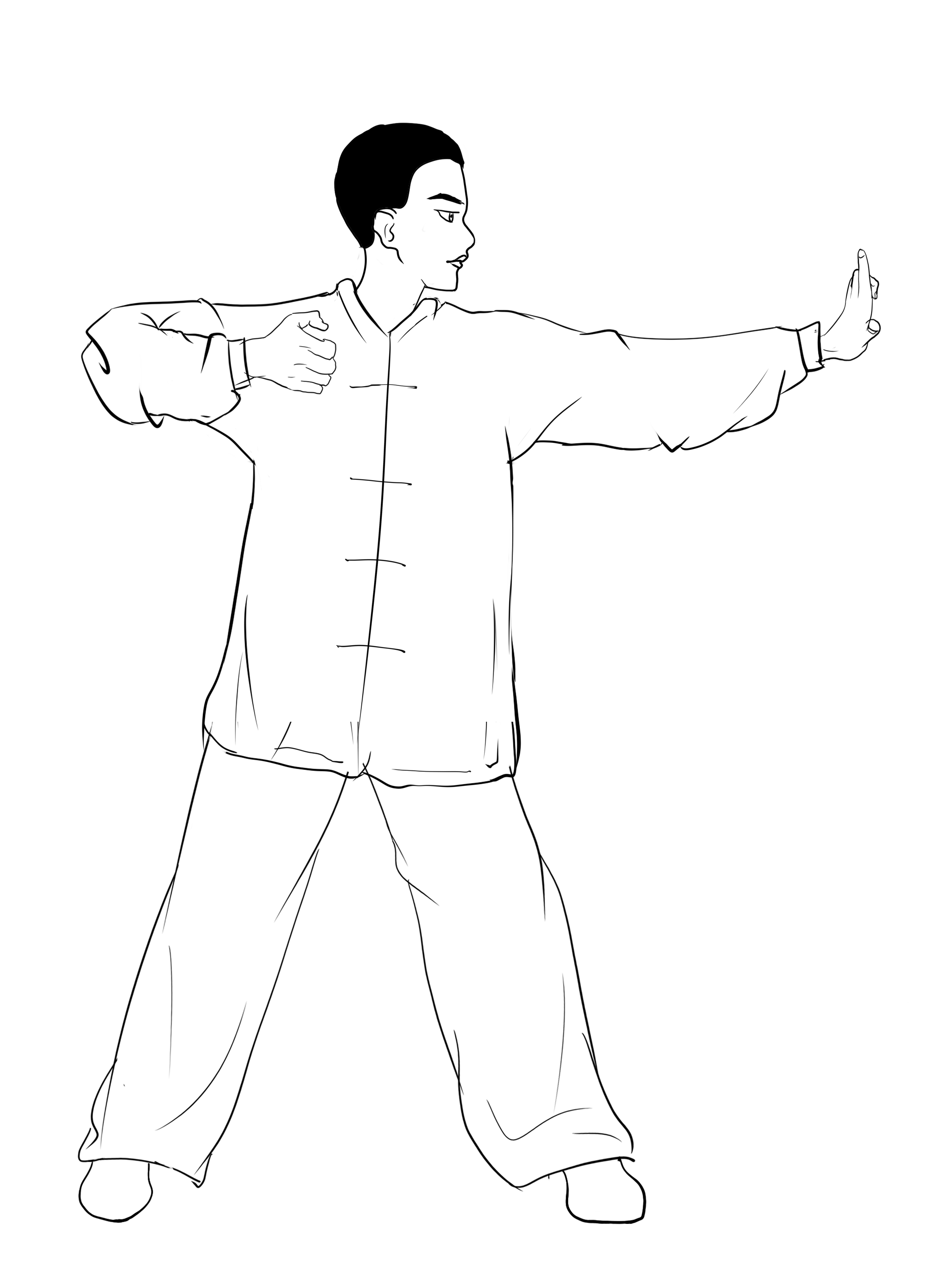 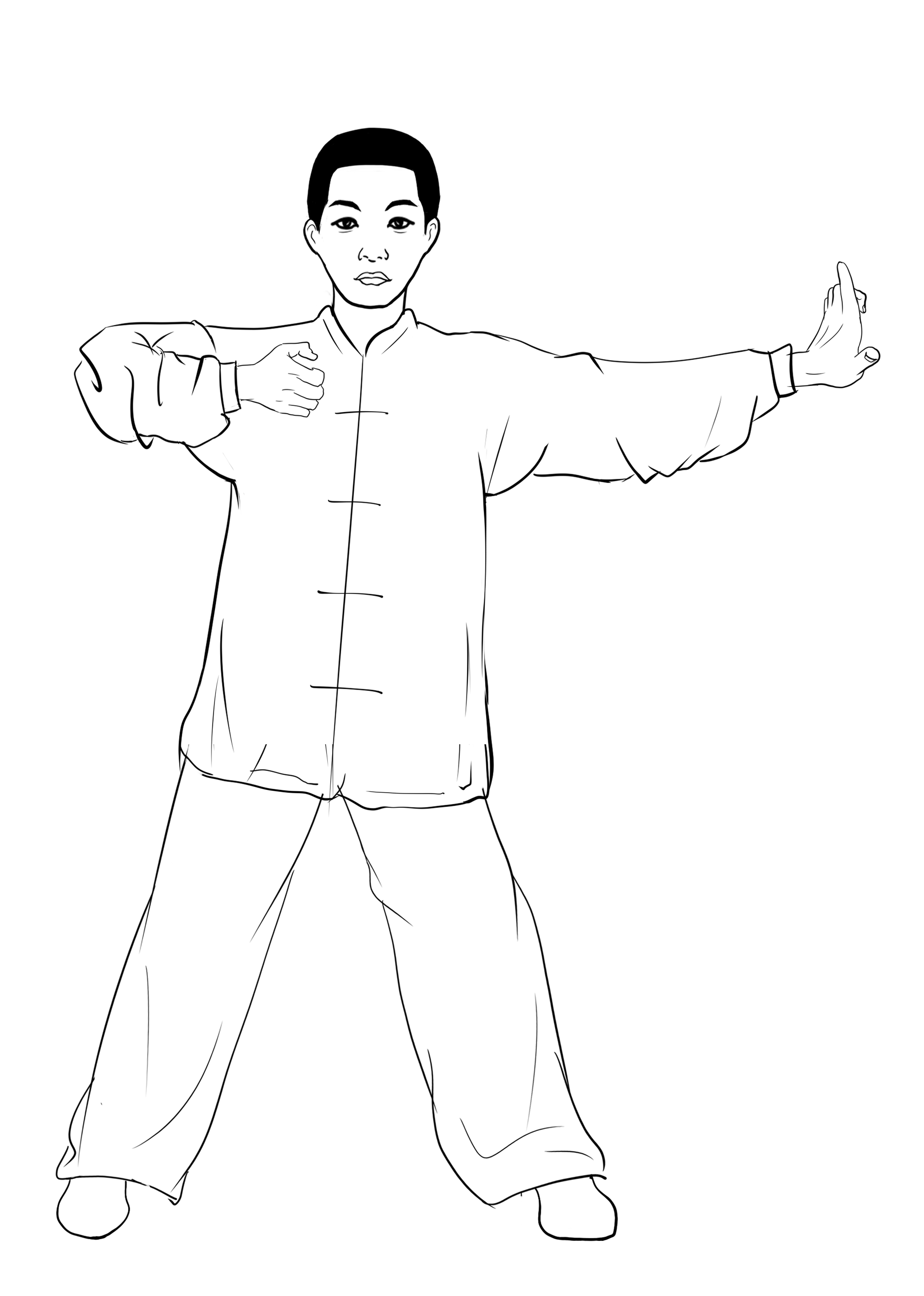**  (d) (e) | (2) Bilateral arm flexion and abduction maneuver. Raise both arms to the level of the upper chest with the palms facing inward, crossing the wrists so that the left wrist is positioned anteriorly and the right wrist posteriorly (b). Form loose fists with both hands, extending the left thumb and index finger while simulating bowstring traction with the right hand (c). Abduct the left arm laterally to full extension while turning the head to the left, keeping the gaze fixed on the tip of the left index finger. At the same time, apply traction with the right arm in the opposite direction, maintaining both arms at the same height (d). Finally, return the head to a neutral position (e). |
| **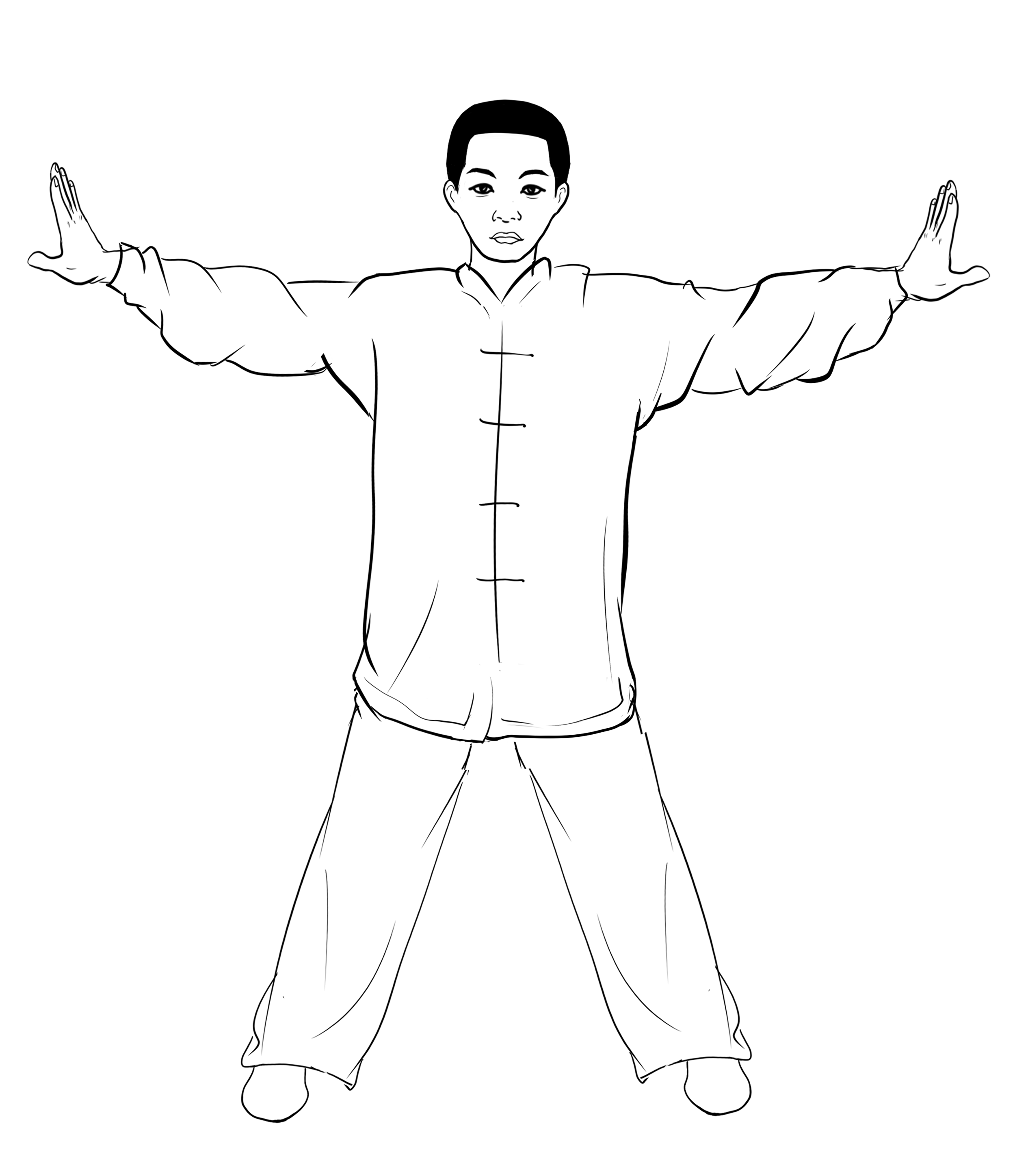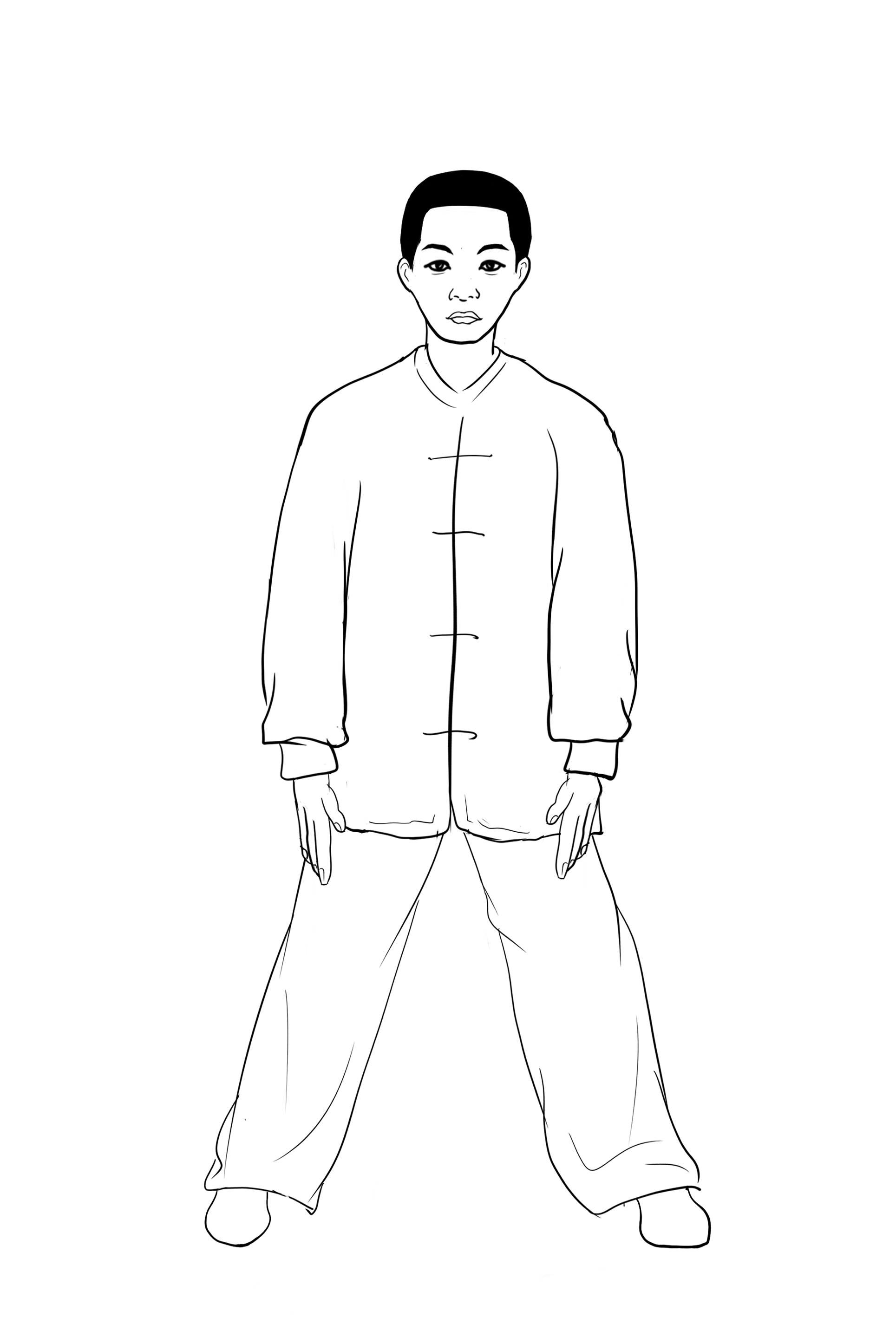**  (f) (g) | (3) Bilateral upper limb return phase. Keep the right arm extended in the coronal plane while maintaining both arms at the same height. Open both hands with the palms extended and the fingers aligned vertically (f). Lower the arms in a controlled manner through shoulder adduction and elbow extension, returning to the initial standing posture(g). |
| The right and left movements are the same but opposite in direction. Both the left and right movements completed is been counted as one time. This movement should be repeated for 3 times. | |

Step 3. Holding one arm aloft to regulate the functions of the spleen and stomach

| **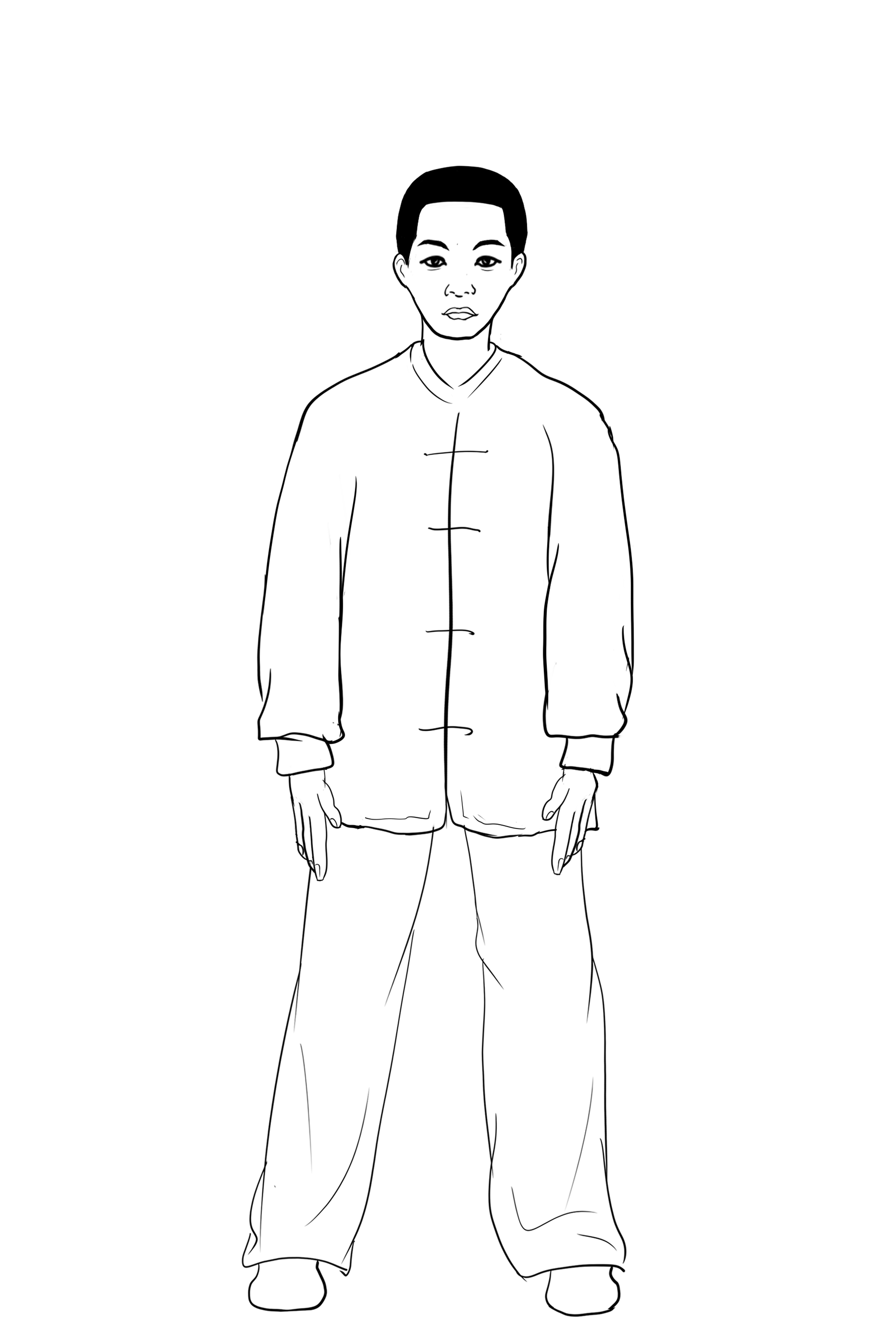**  (a) | (1) Preparatory posture. The same as (1) in Step 1. |
| --- | --- |
| **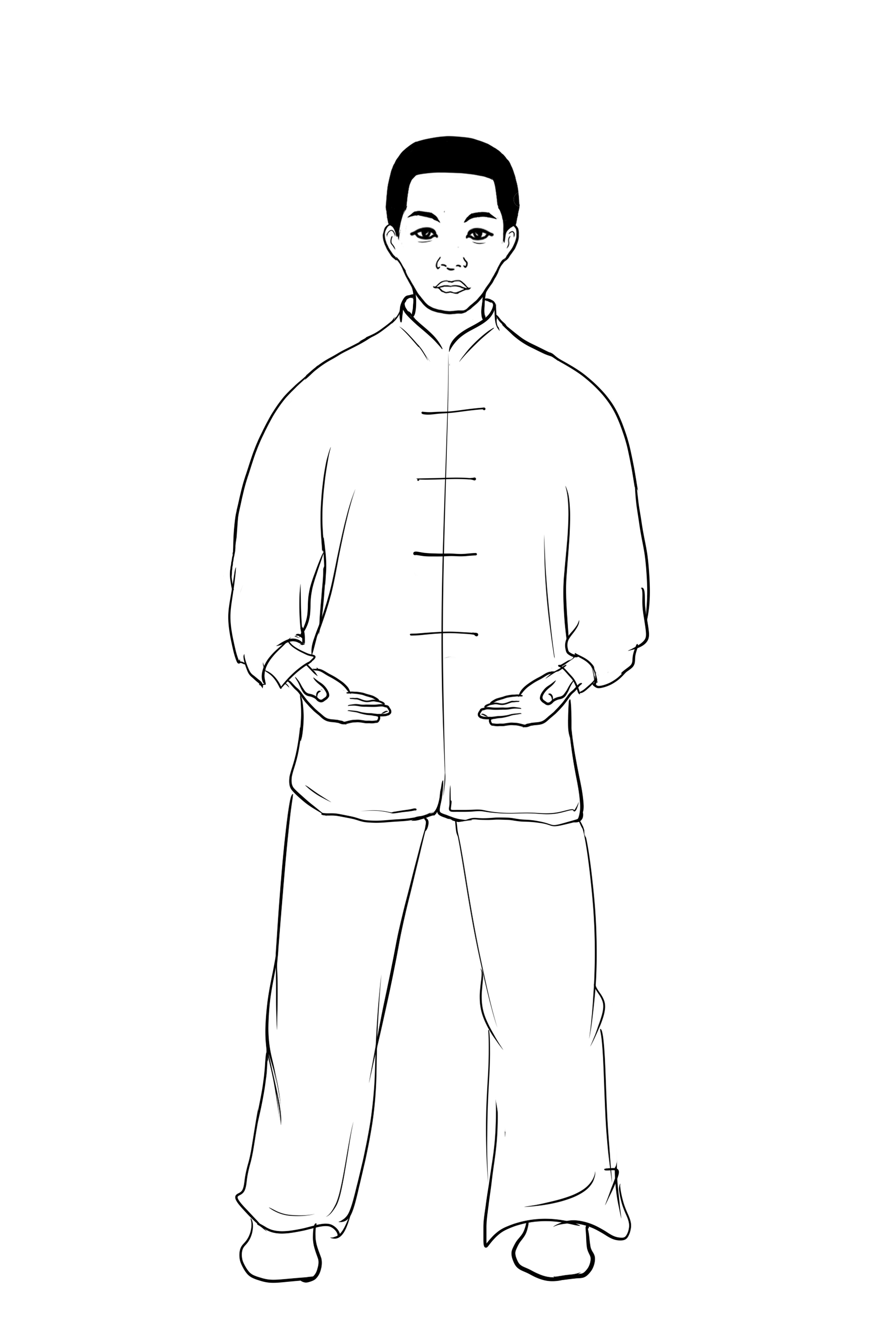**  (b)  **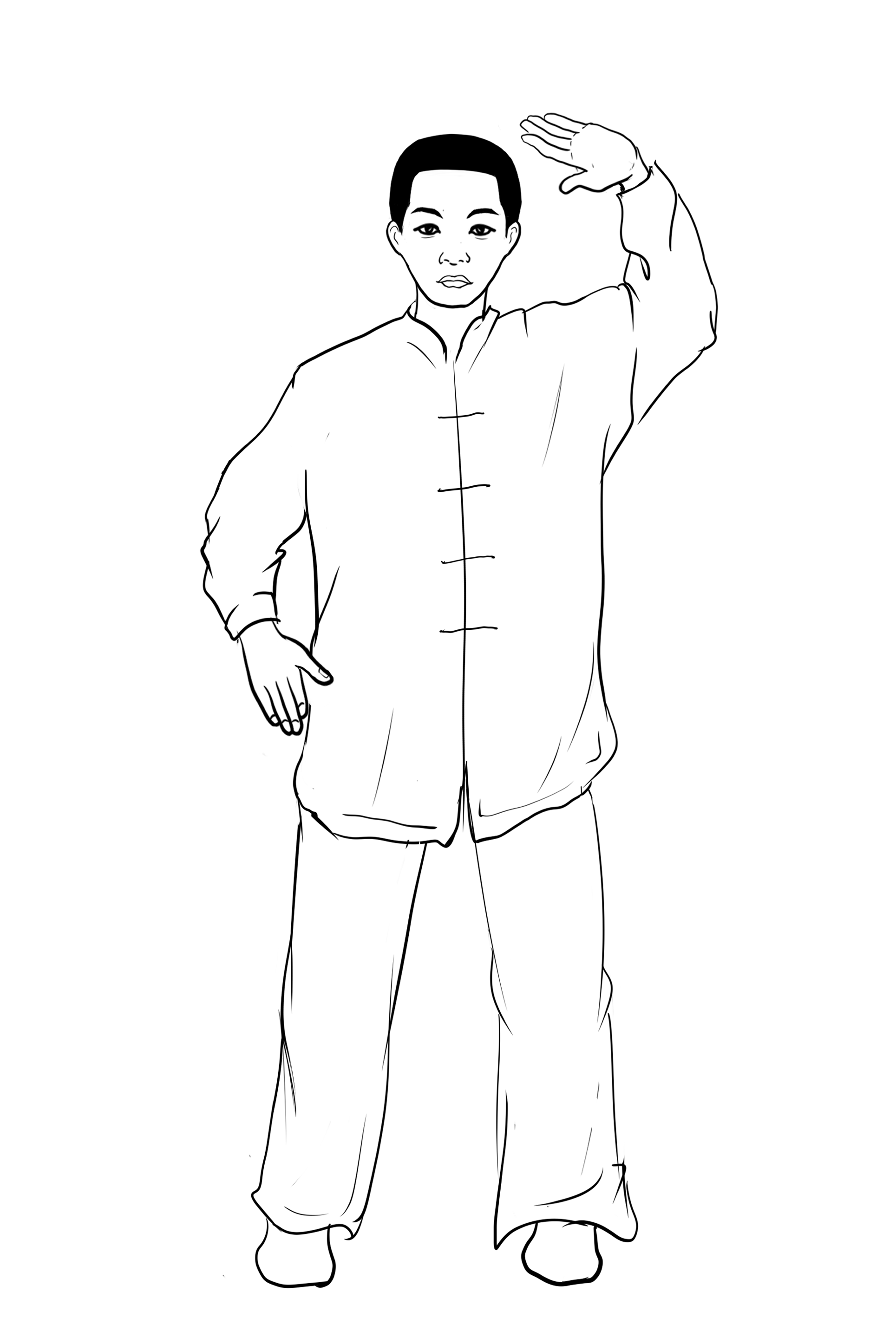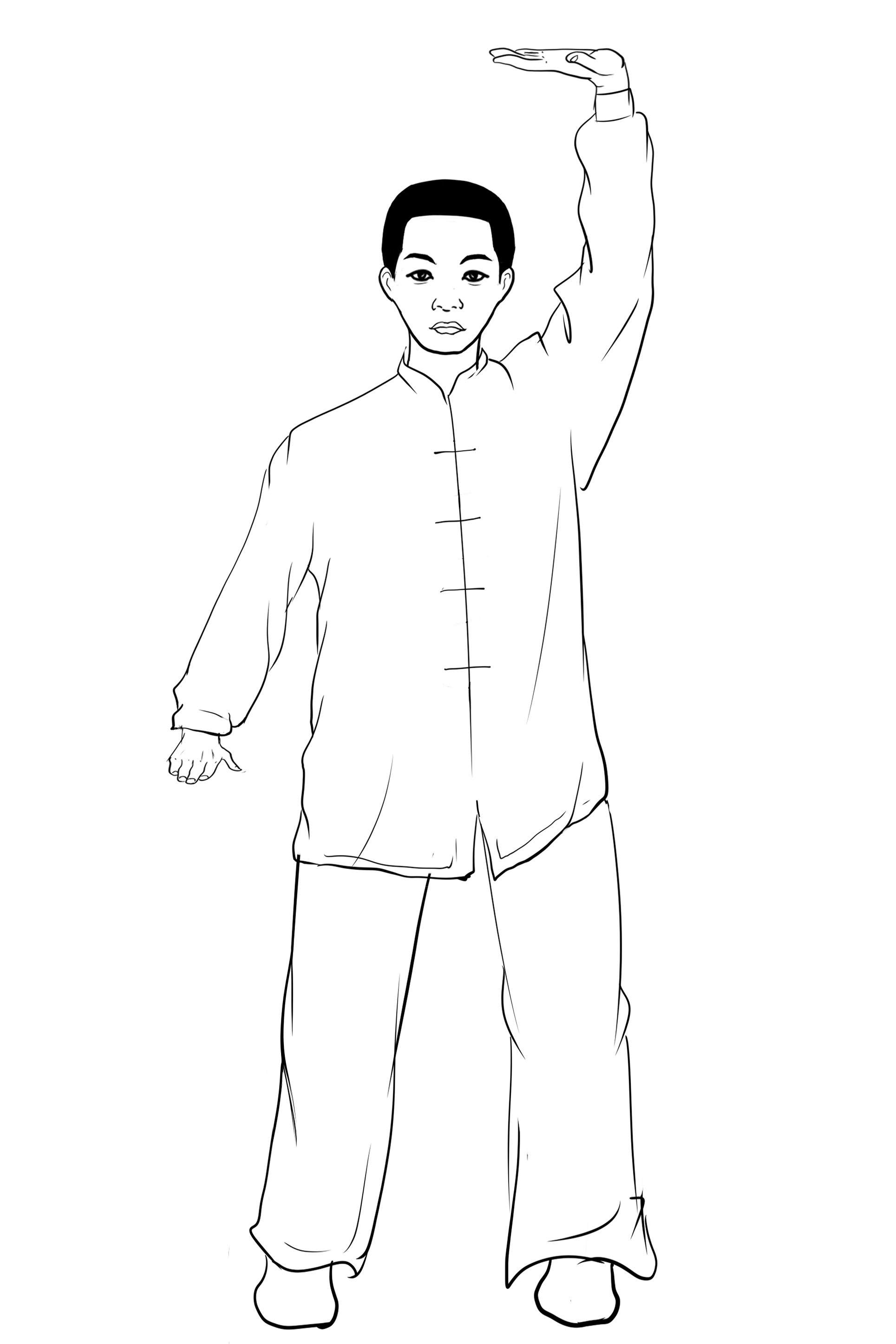**  (c) (d) | (2) Bilateral upper limb asymmetric press maneuver. With the elbows slightly flexed, place both hands in front of the lower abdomen with the palms facing upward (b). Slowly raise the left arm laterally to its maximal range while rotating the wrist so that the palm faces upward and the fingertips point to the right. At the same time, rotate the right wrist so that the palm faces downward with the fingertips directed forward, pressing the right arm downward to its maximal range (c). Conclude the movement by exerting upward pressure with the left arm and simultaneous downward pressure with the right arm (d). |
| **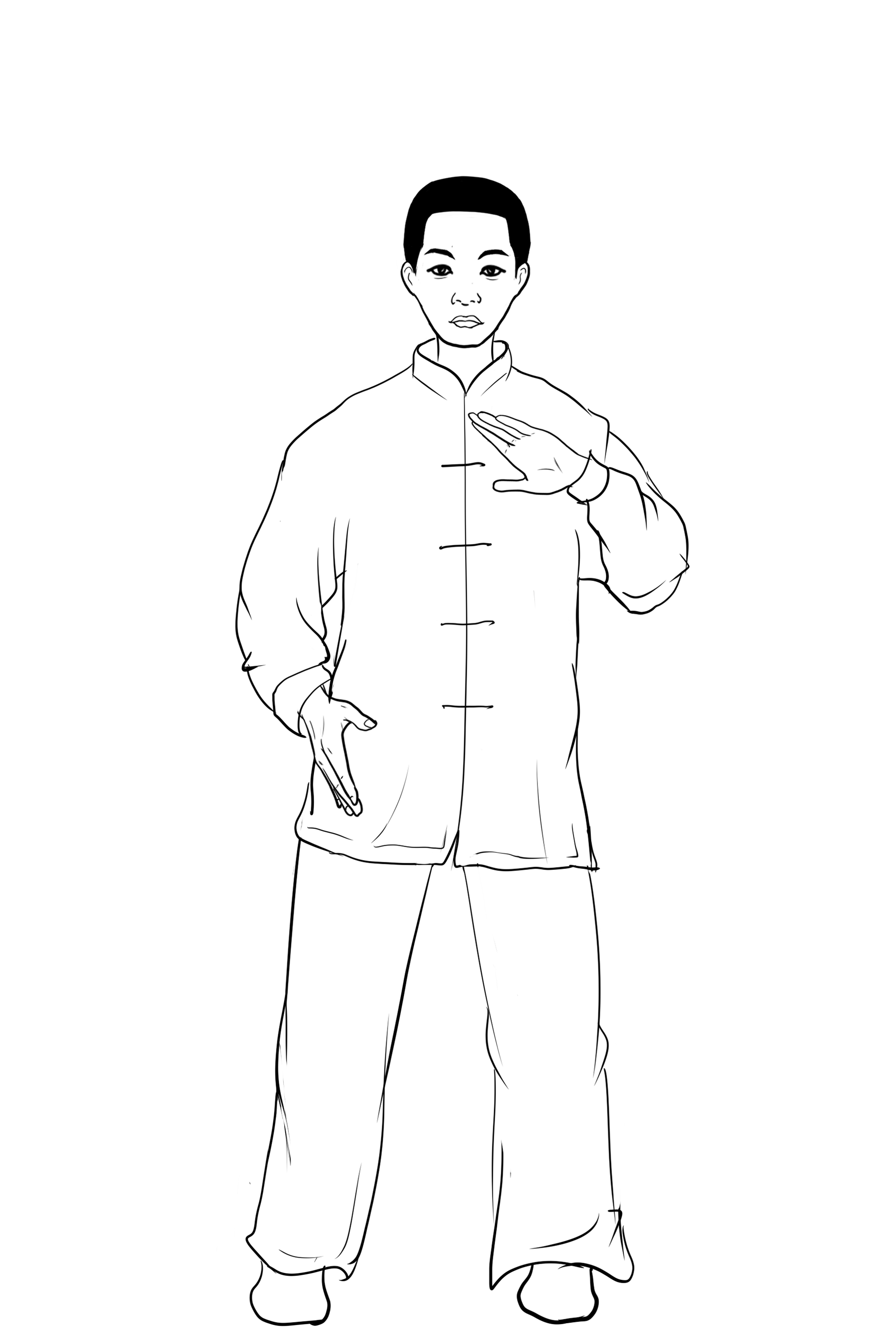 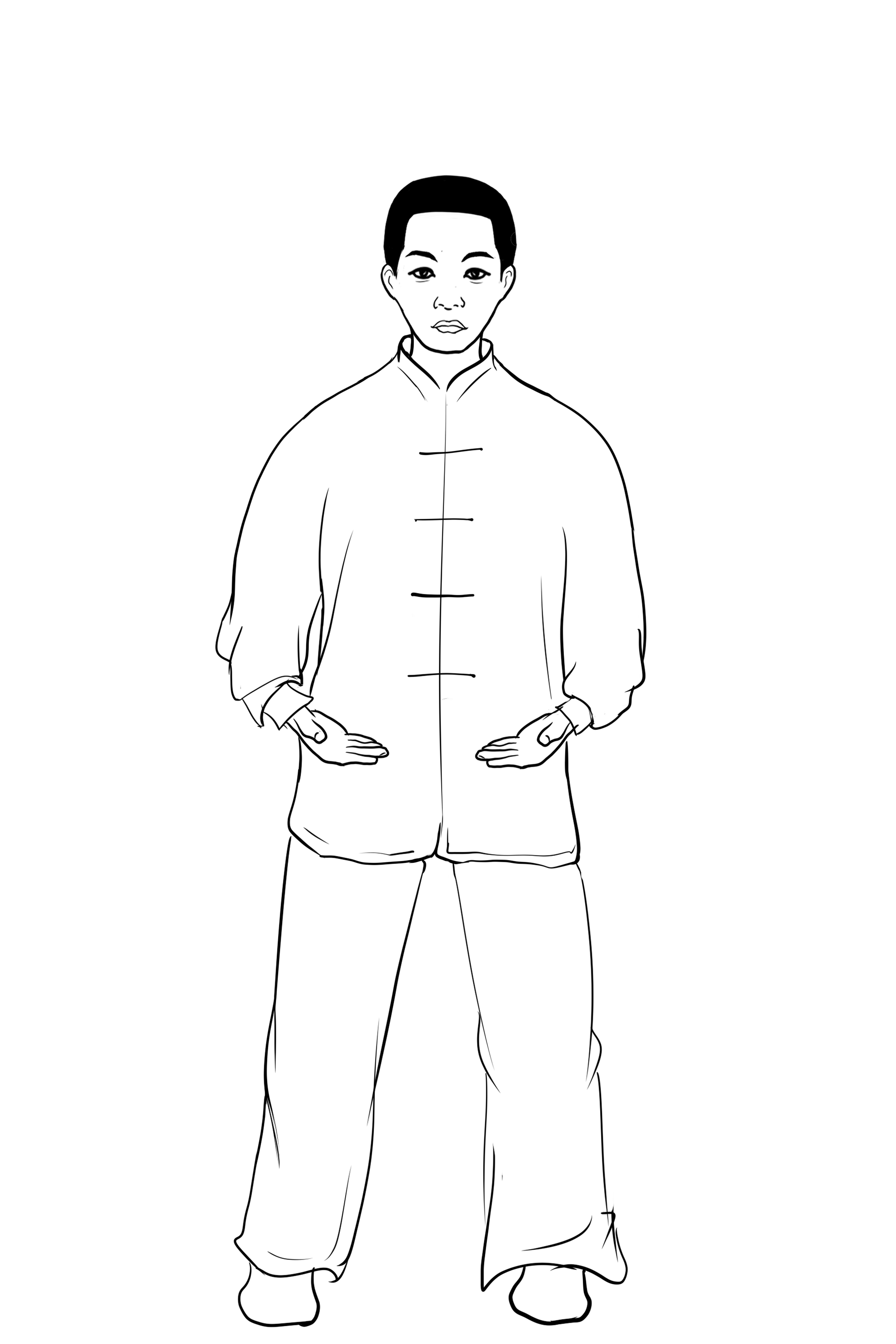**  (e) (f)  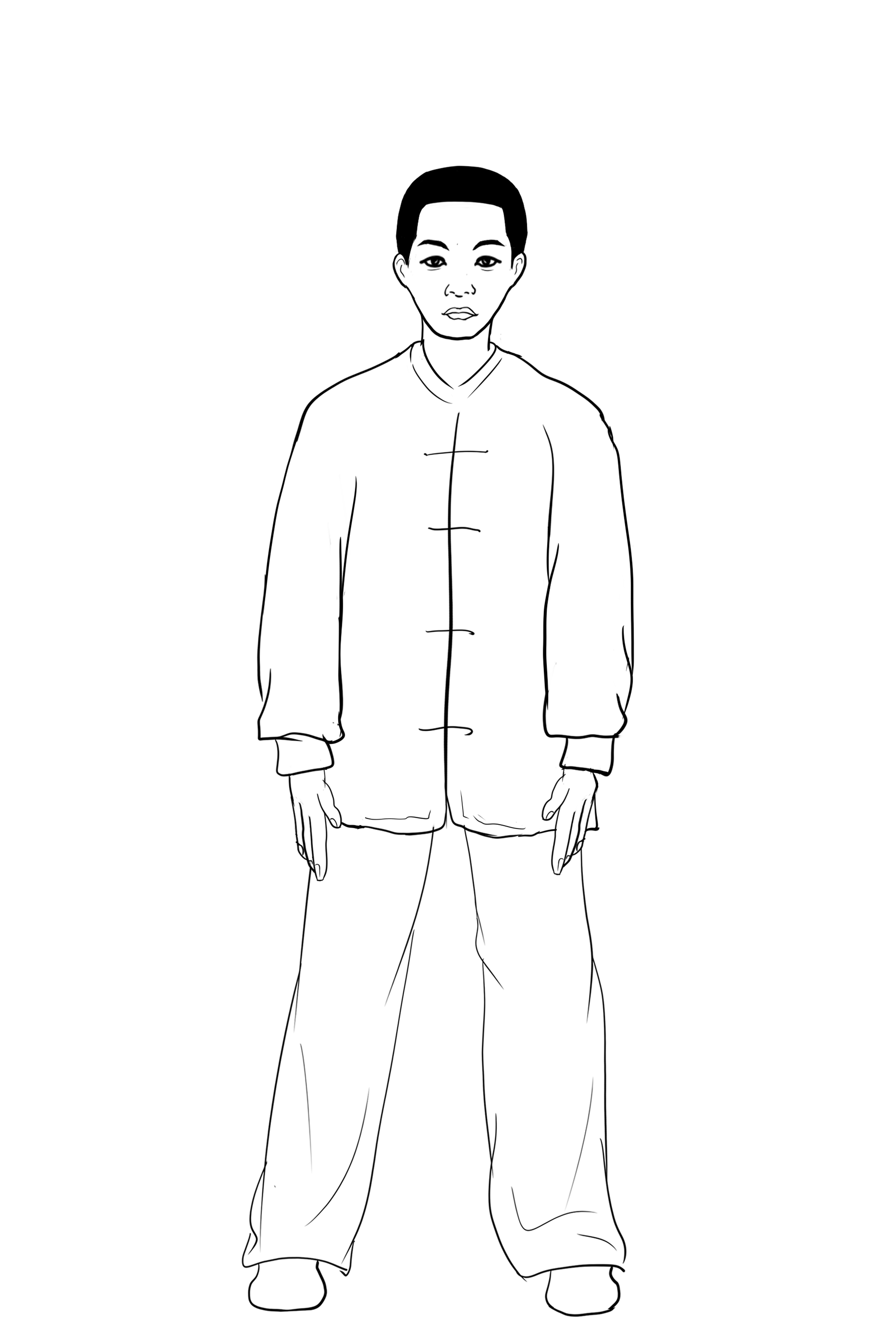  (g) | (3) Bilateral upper limb returns with reciprocal motion. Rotate the left palm downward while slowly lowering the arm and simultaneously rotate the right palm upward while gradually raising the arm (e). Flex both elbows to bring the hands in front of the lower abdomen with the palms facing upward (f). Press both palms gently downward to conclude the movement and return to the standing posture (g). |
| The right and left movements are the same but opposite in direction. Both the left and right movements completed is been counted as one time. This movement should be repeated for 3 times. | |

Step 4. Looking backwards to prevent sickness and strain

| 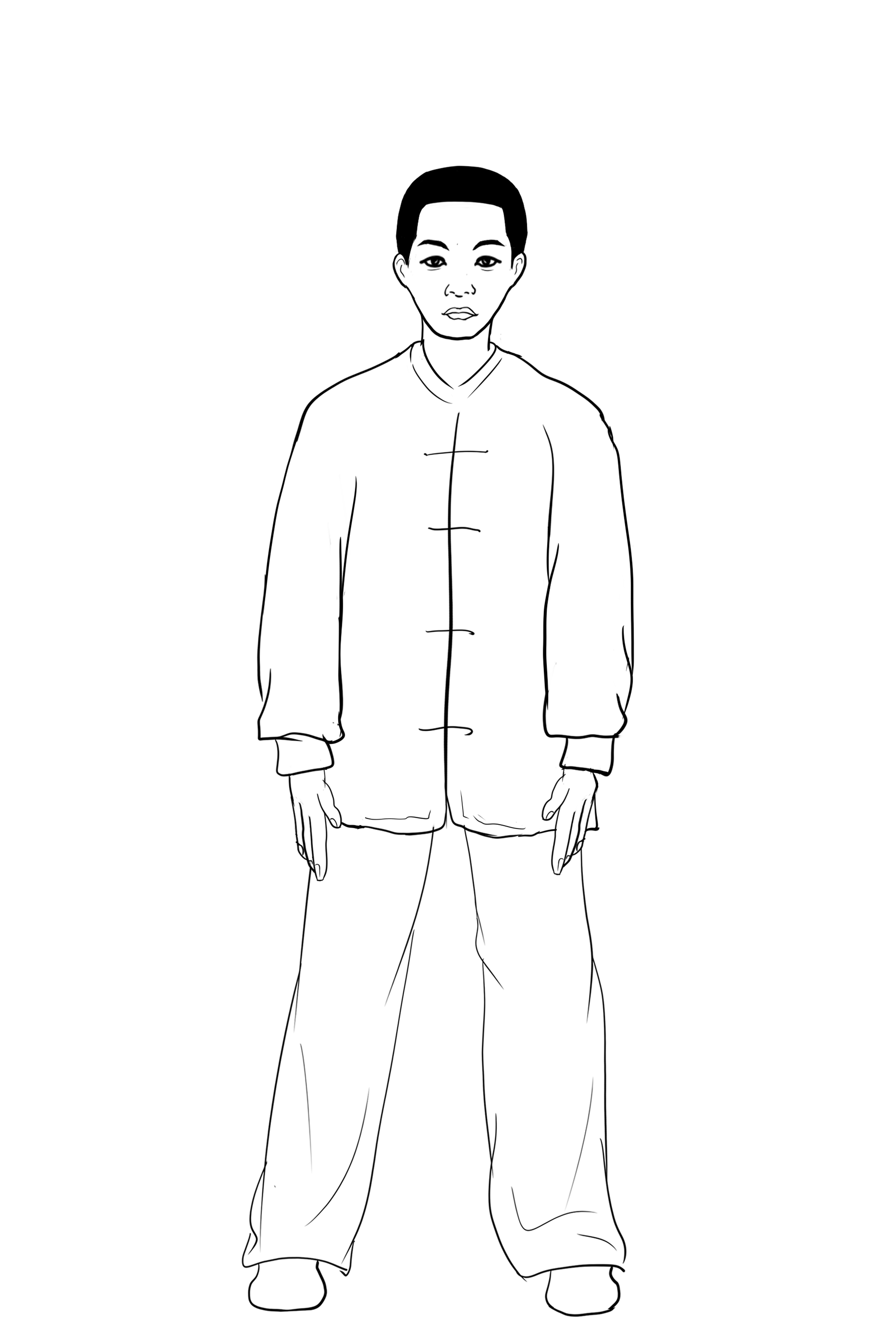  (a) | (1) Preparatory posture. The same as (1) in Step 1. |
| --- | --- |
| 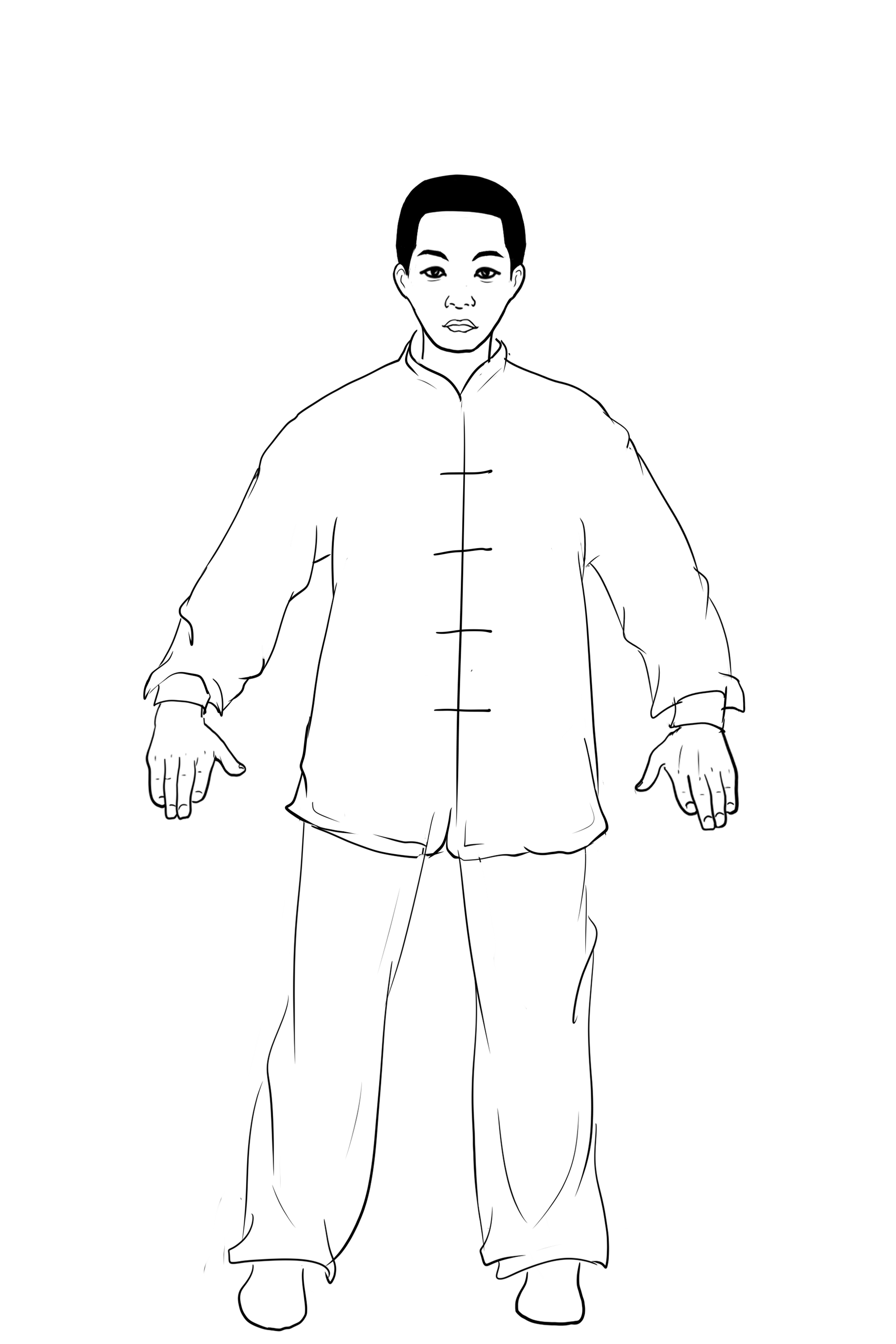 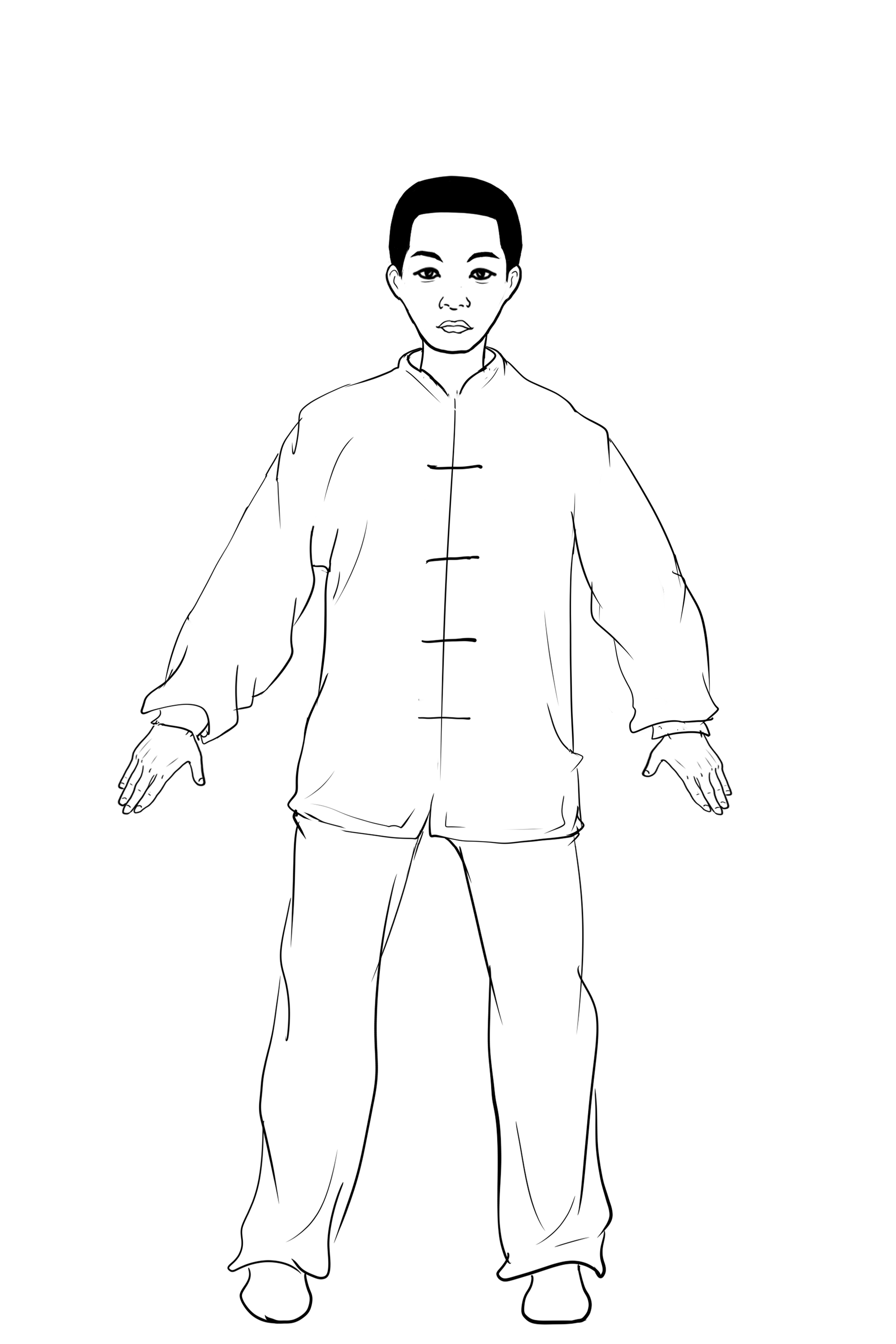  (b) (c)  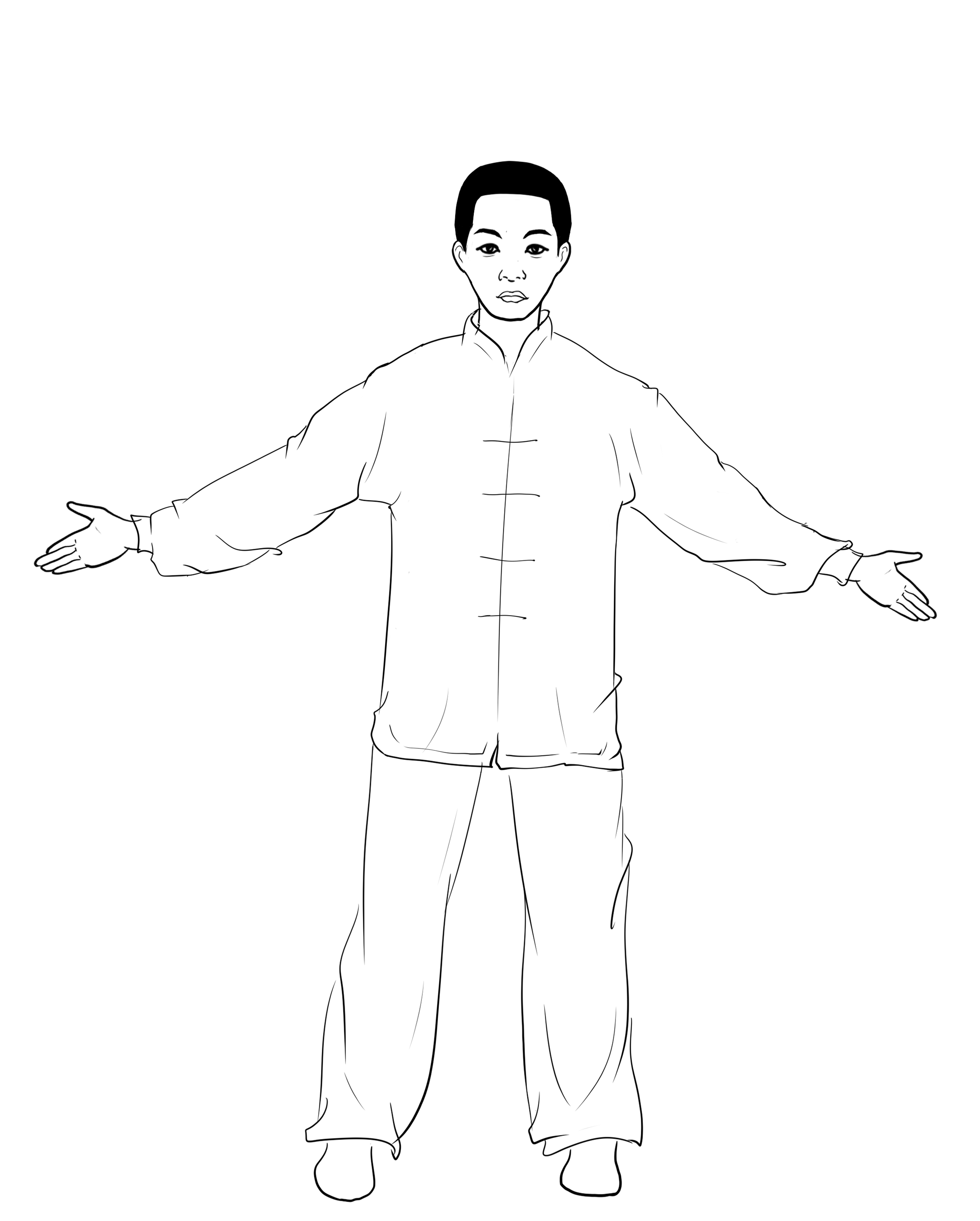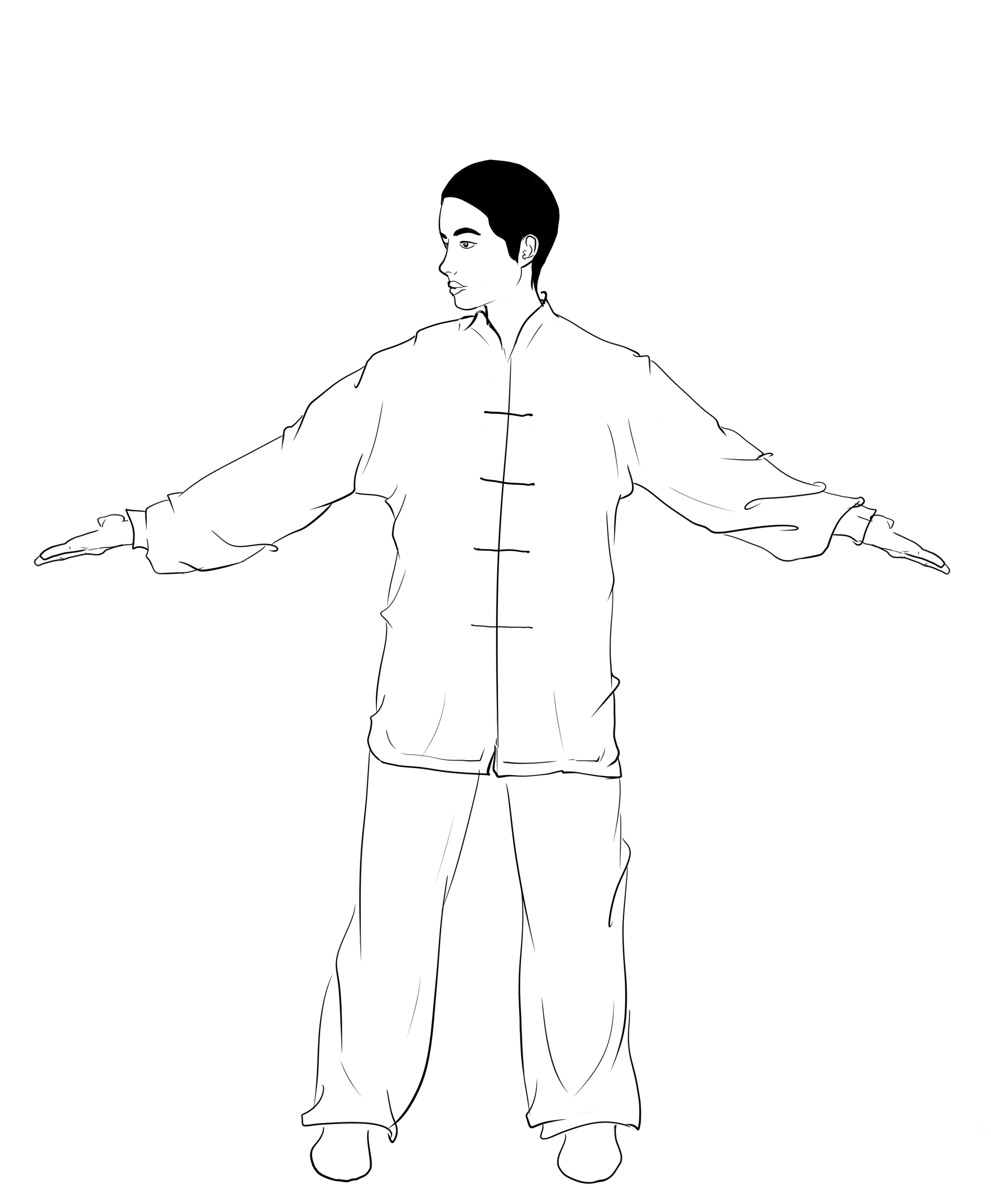  (d) (e) | (2) Cephalic rotation with bilateral upper limb synchronization. Straighten the legs while rotating both arms so that the palms face backward (b). Slowly move the arms laterally to shoulder level with the palms lifted to the height of the hips (c). Externally rotate the shoulders, elbows, and wrists in a coordinated manner (d). With the palms still facing backward, rotate the head to the right as far as comfortable, directing the gaze toward the fingertips (e). |
| 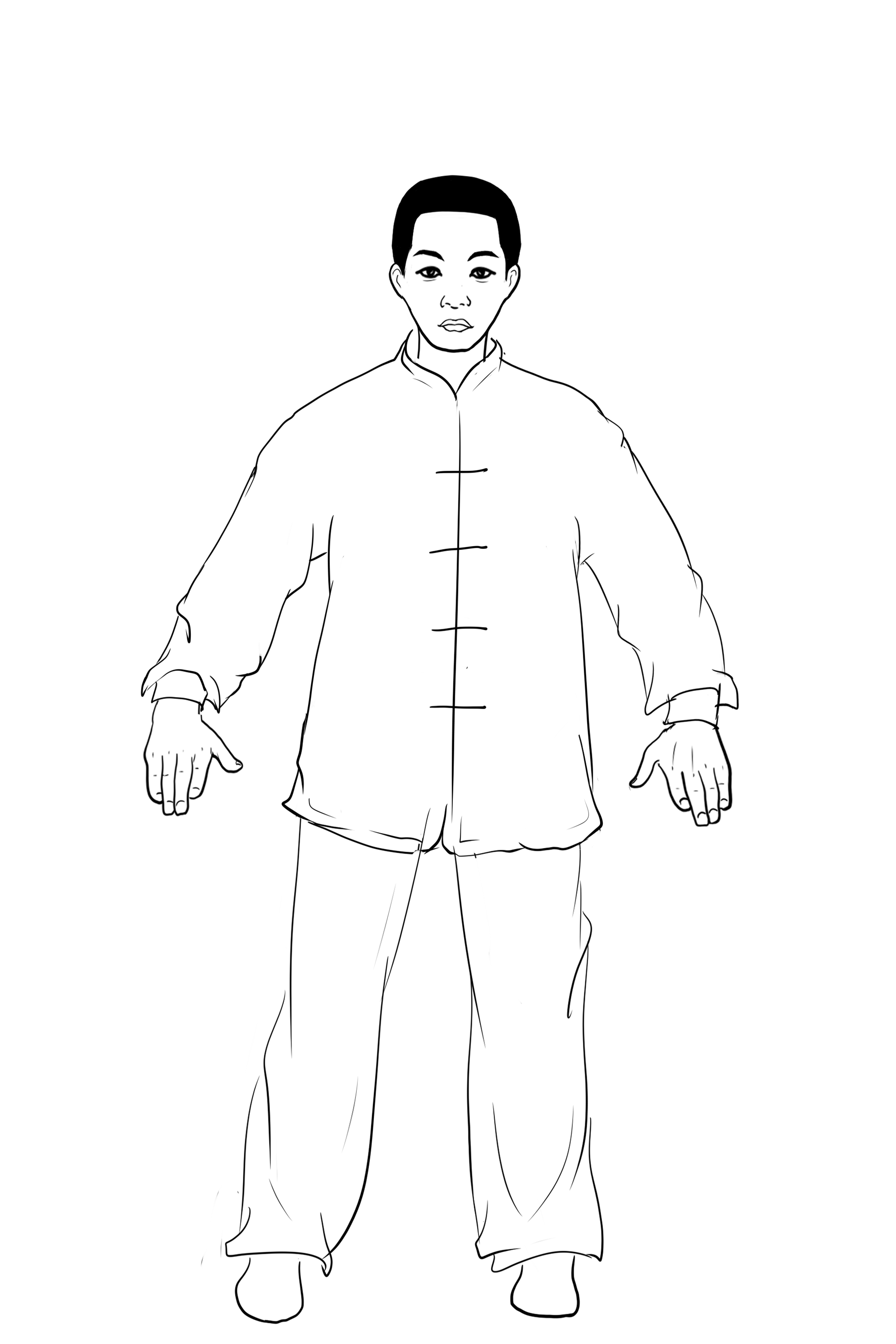 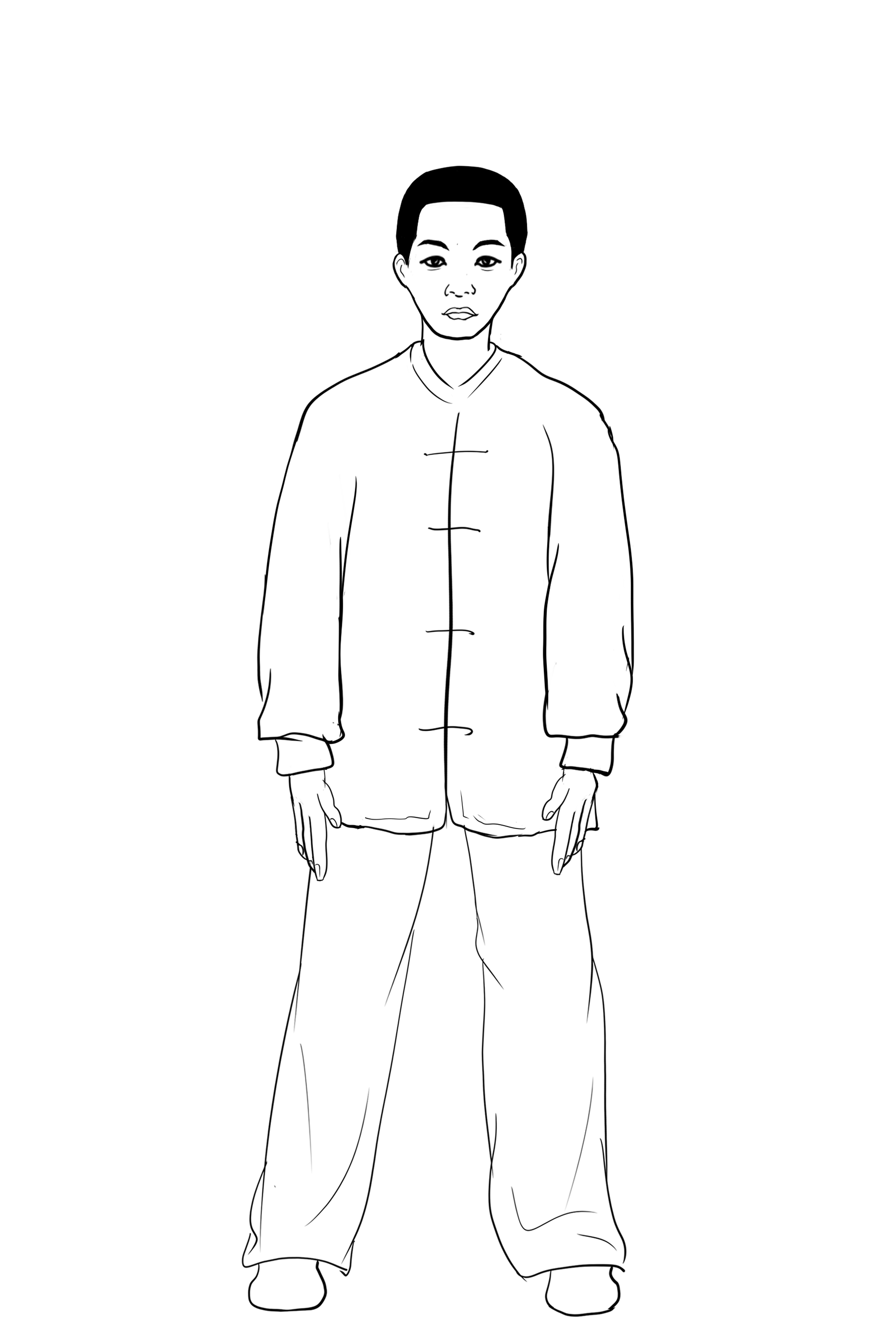  (f) (g) | (3) Bilateral upper limb. Return the head to a neutral position while rotating the shoulders, elbows, and wrists inward. Bring both arms back to the sides of the body with the palms facing downward (f). Conclude by resuming the initial standing posture (g). |
| The right and left movements are the same but opposite in direction. Both the left and right movements completed is been counted as one time. This movement should be repeated for 3 times. | |

Step 5. Thrusting the fists and making the eyes glare to enhance strength

| 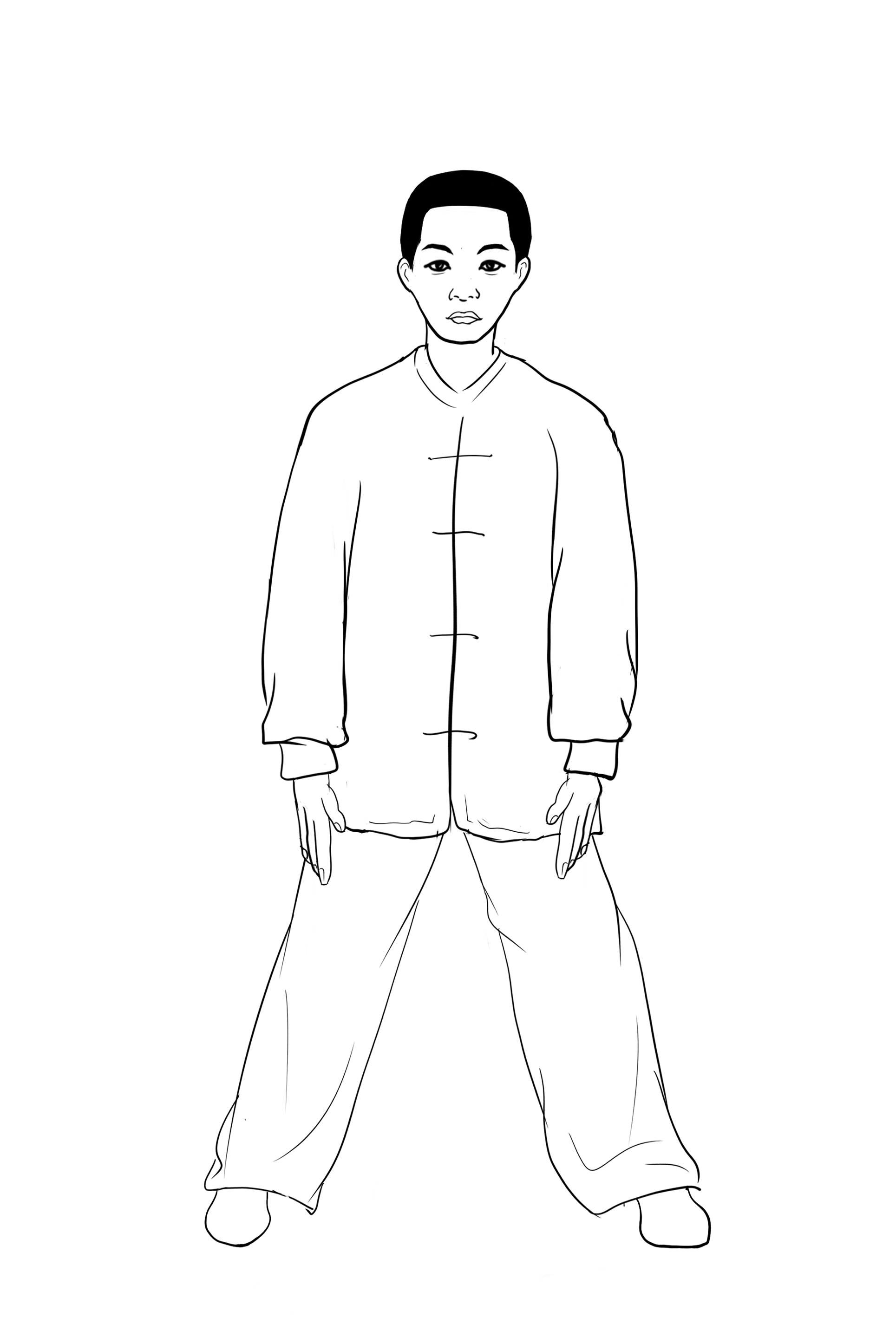  (a) | (1) Preparatory posture. The same as (1) in Step 2. |
| --- | --- |
| 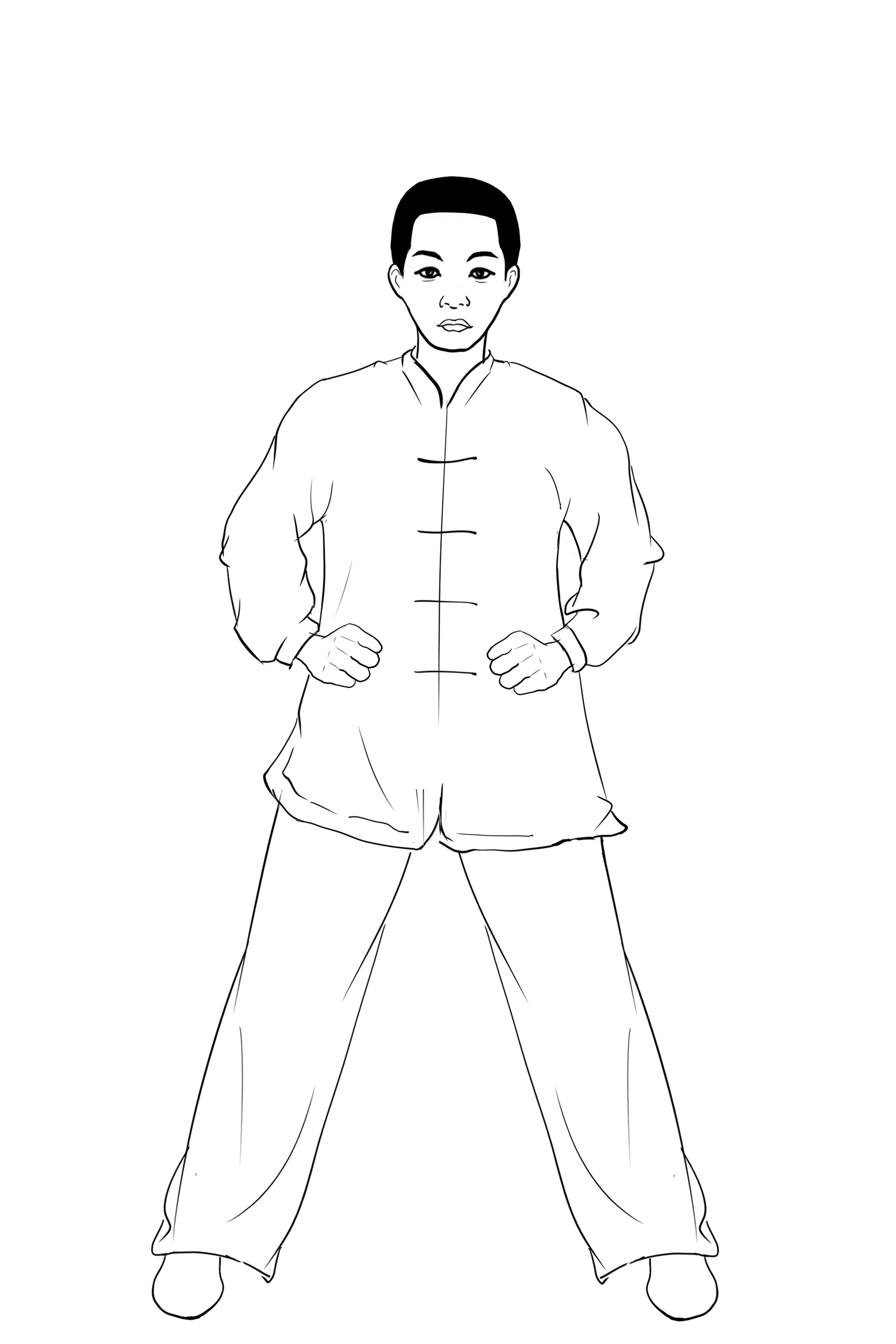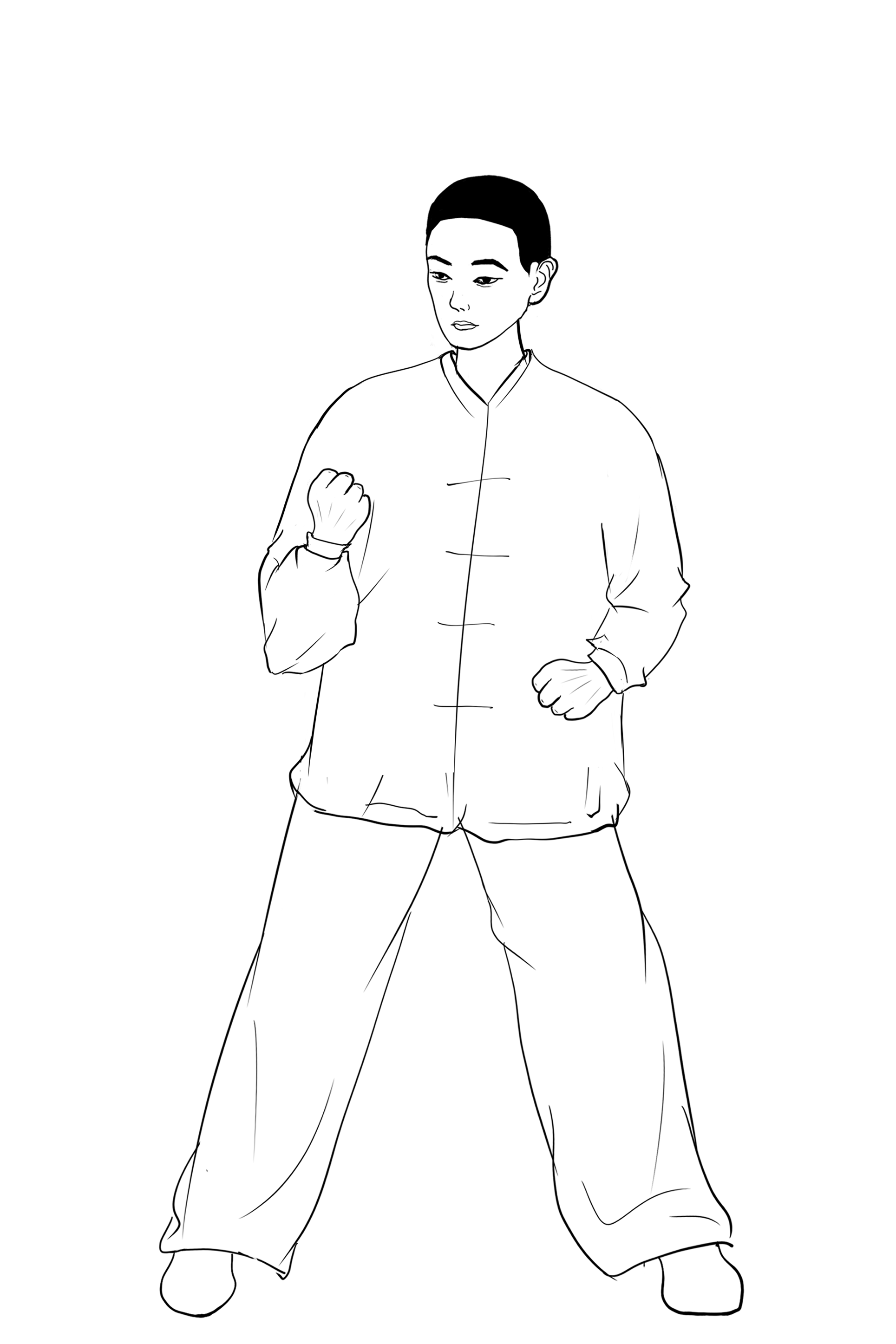  (b) (c)  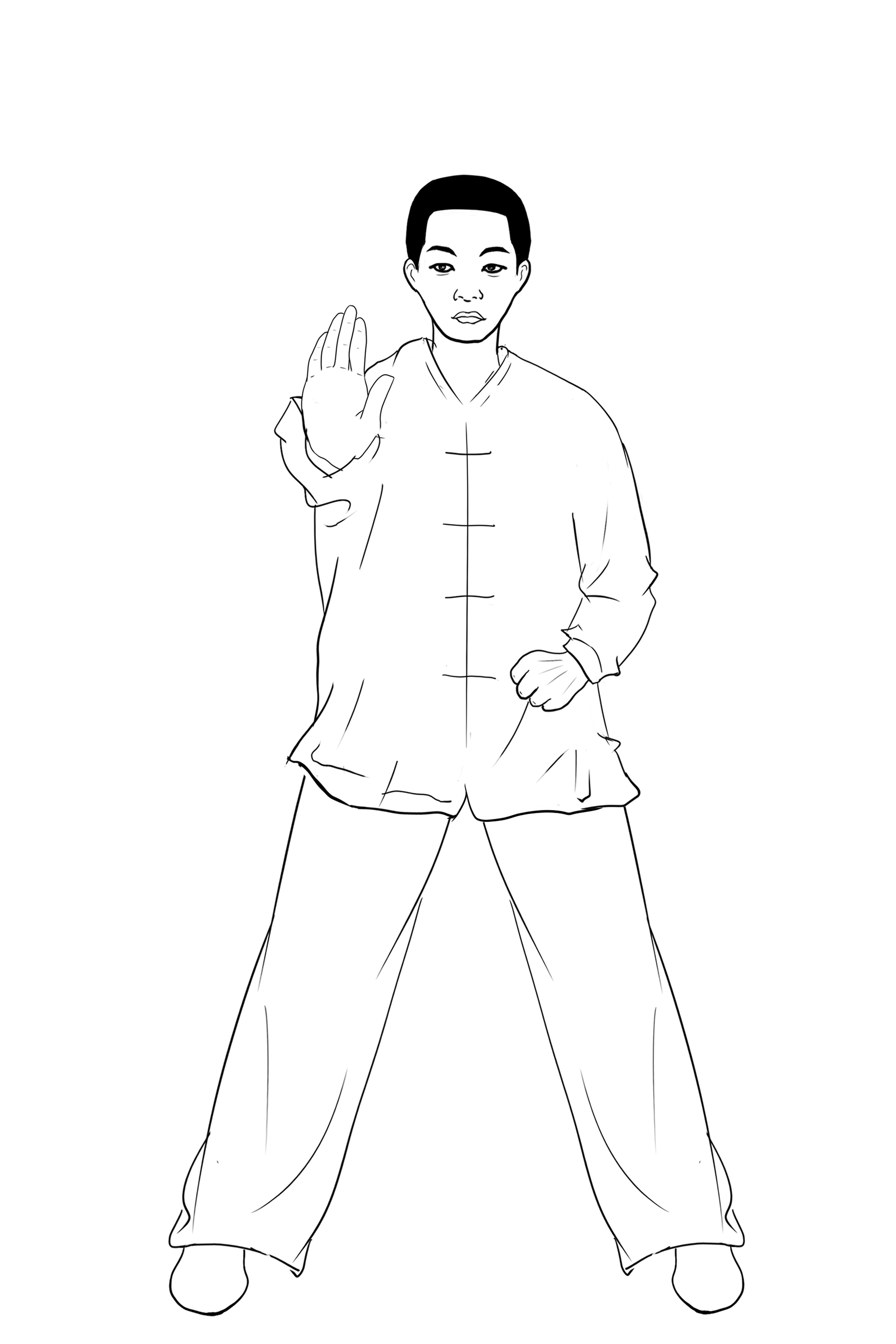 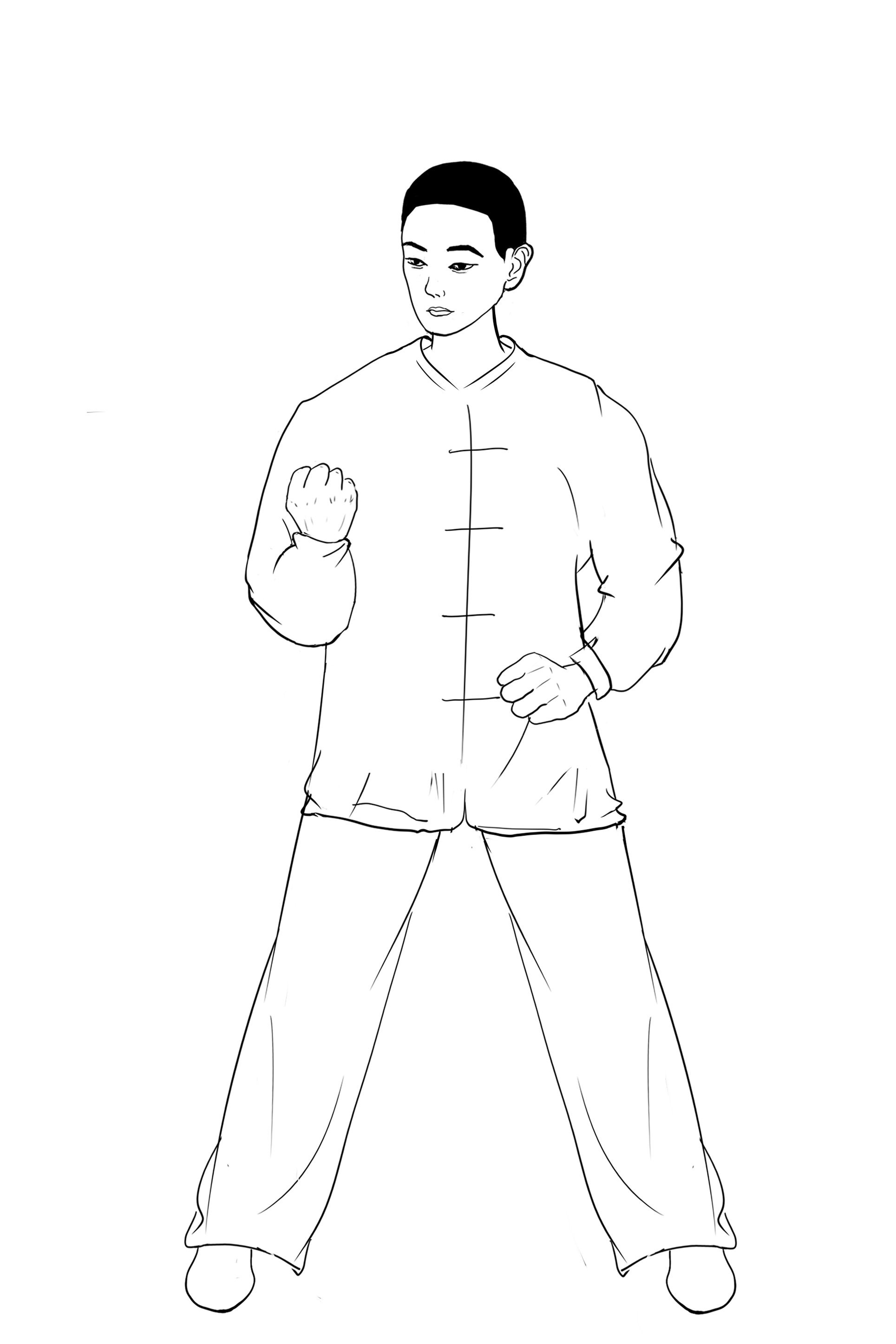  (d) (e) | (2) Unilateral boxing maneuver. Raise both hands to the sides of the waist with the palms facing inward, forming fists while slightly bending the knees into a squat position, ensuring that the knees do not extend beyond the toes (b). Perform a forward thrust with the right fist, gradually lifting it to shoulder height while keeping the gaze fixed on the moving hand (c). Fully extend the right arm, then open the hand with the fingers together and the palm facing forward, perpendicular to the ground (d). Rotate the wrist so that the palm faces upward, reform a fist, and draw the right arm back toward the side of the torso while maintaining visual focus on the right hand (e). |
| 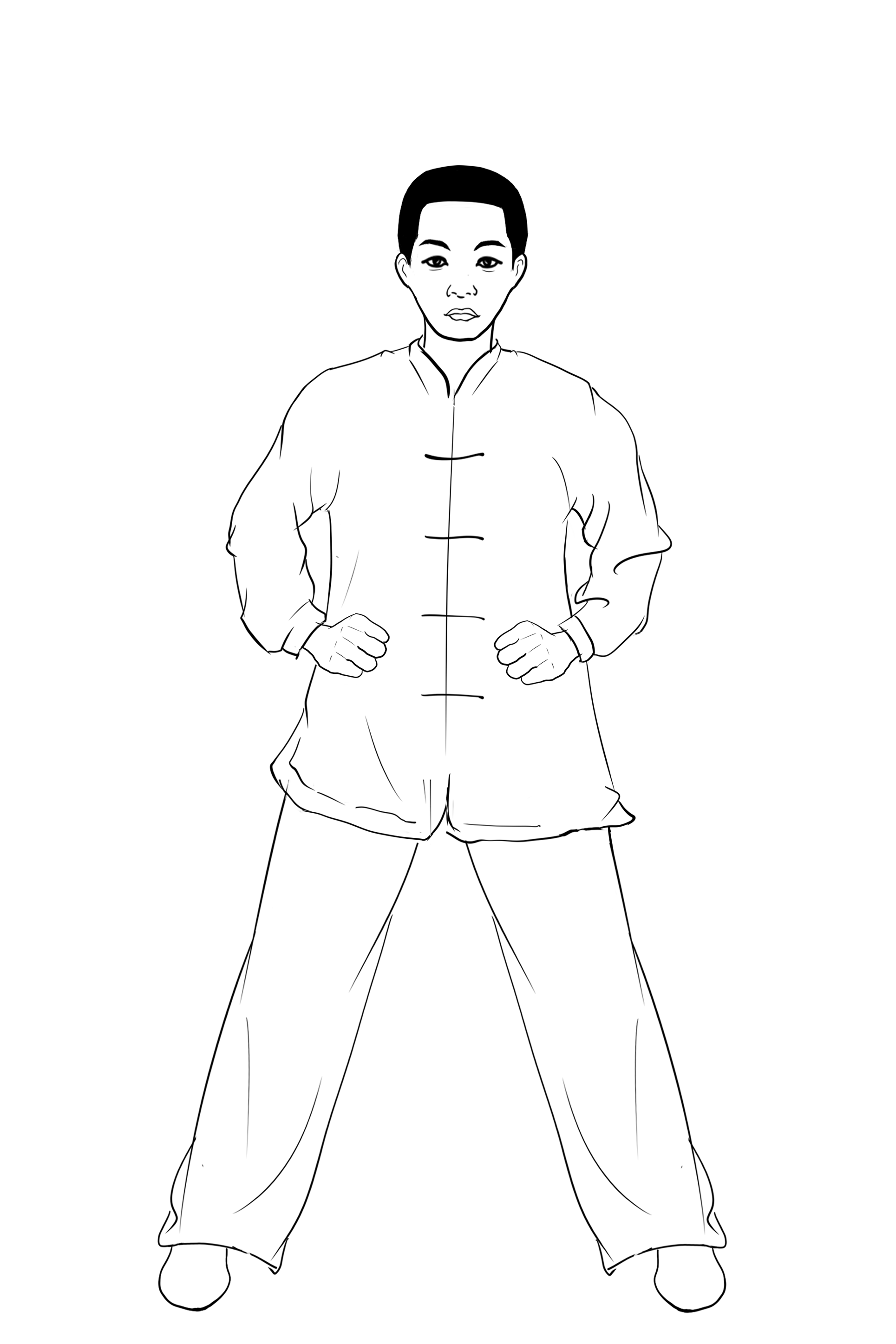 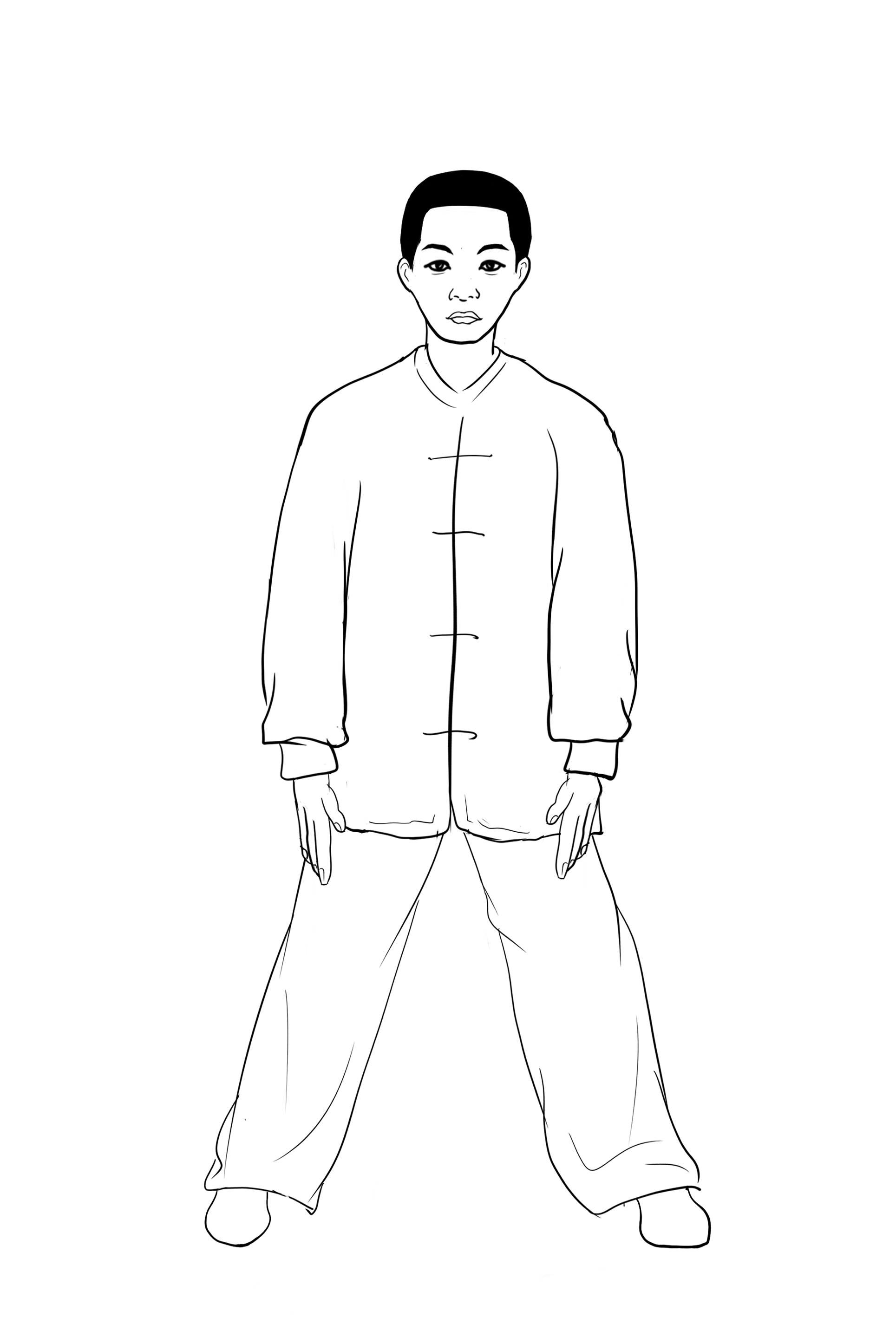  (f) (g) | (3) Bilateral upper limb. Return the head to neutral alignment (f). Open both fists into flat palms with the fingers fully extended and held together. Gradually lower the upper limbs while extending the knees to complete the transition back to the fundamental standing posture (g). |
| The right and left movements are the same but opposite in direction. Both the left and right movements completed is been counted as one time. This movement should be repeated for 3 times. | |
