# Supplementary material for: Baduanjin exercise with or without traditional Chinese tuina therapy for nonspecific chronic neck pain: study protocol for a randomised controlled trial
Source: Front Sports Act Living. 2026 Mar 13;8:1787515. doi: 10.3389/fspor.2026.1787515 (PMC13021794; doi:10.3389/fspor.2026.1787515)
Supplement: Supplementary Document 1 — SPIRIT Checklist. [file table2.doc]

**Step 1.** Local manipulation

| 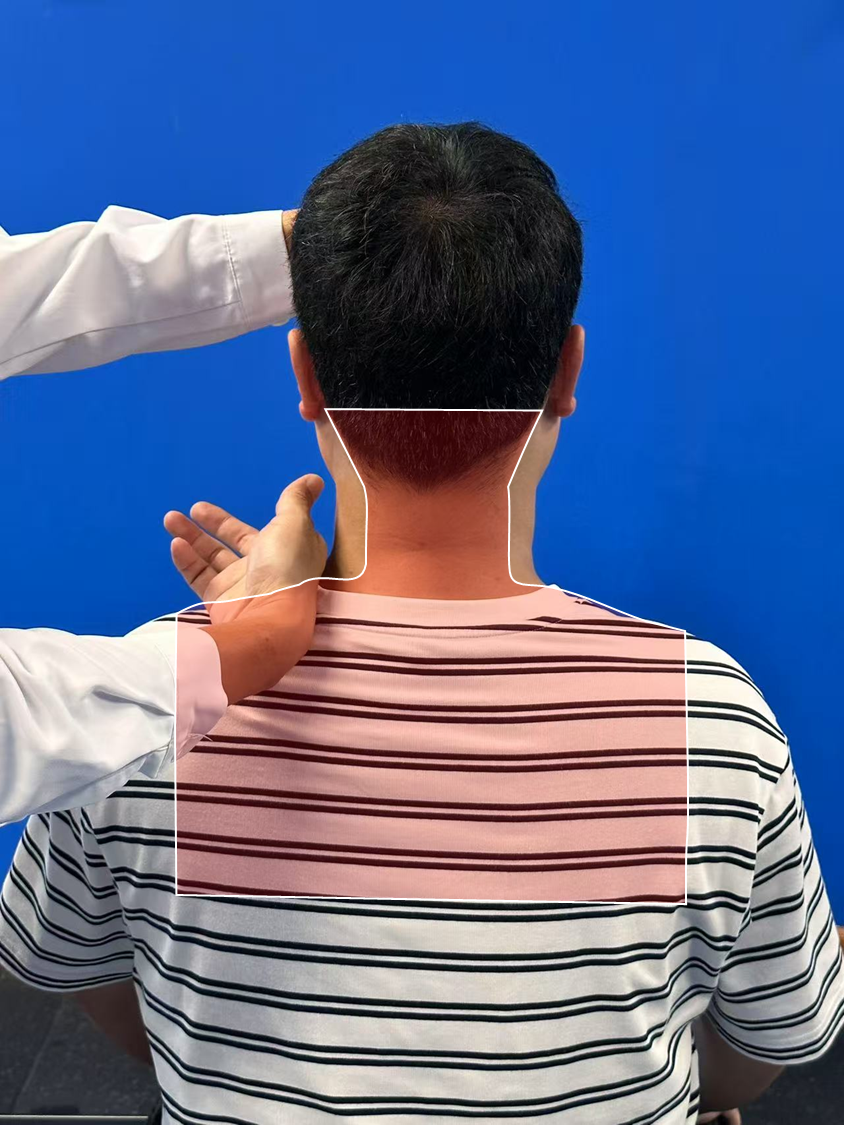  (a) | 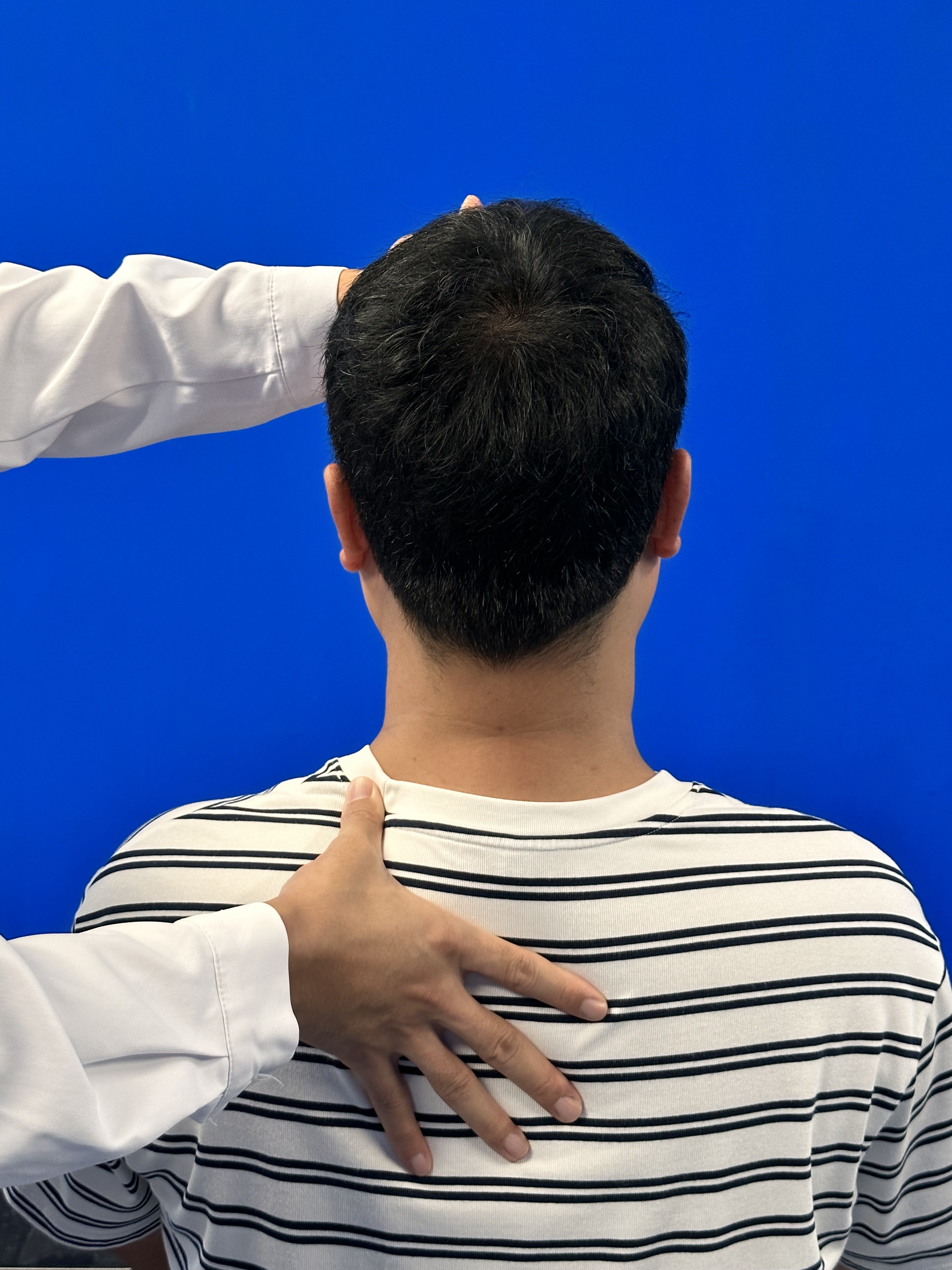  (b) |
| --- | --- |
| 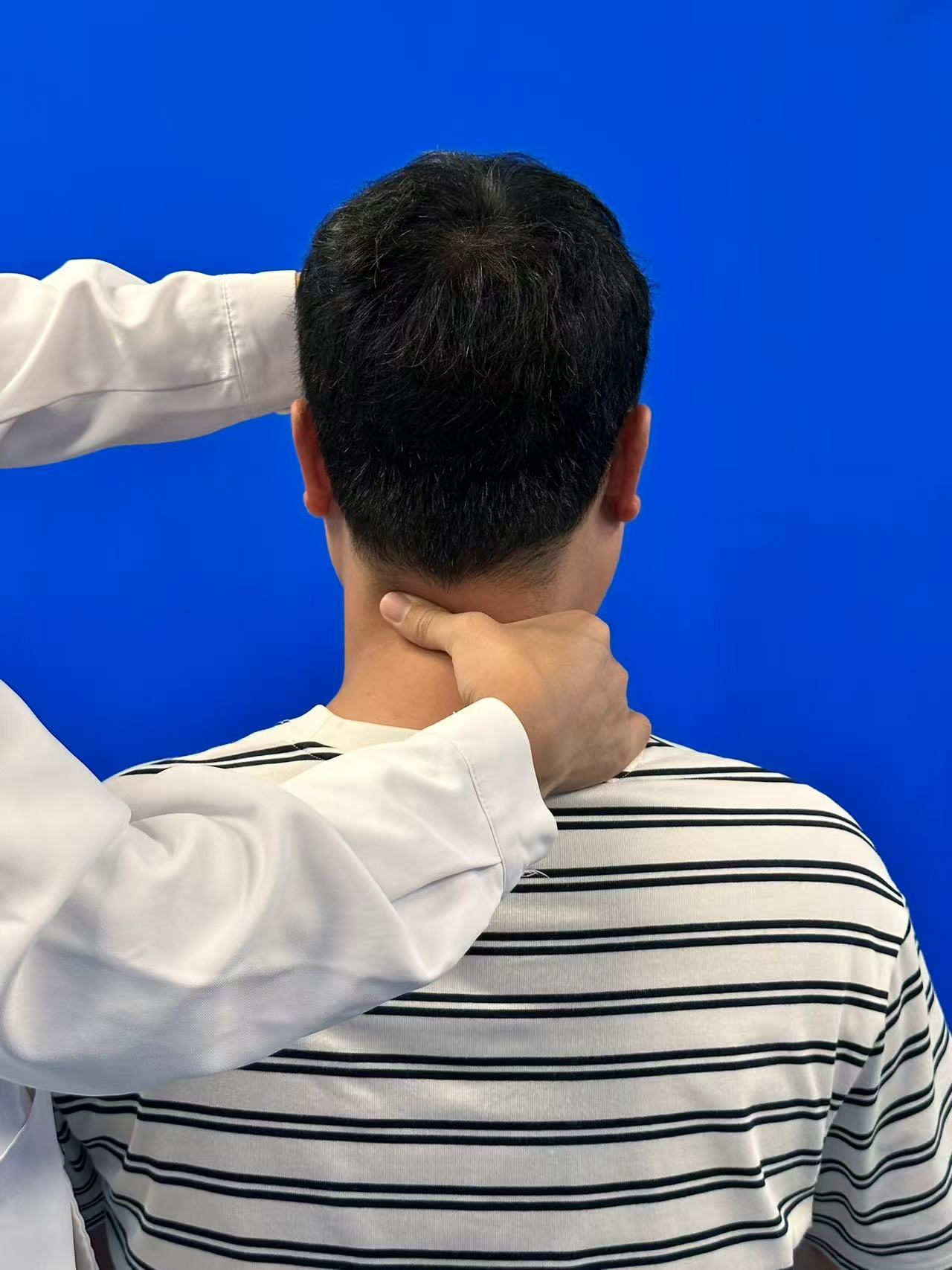  (c) | 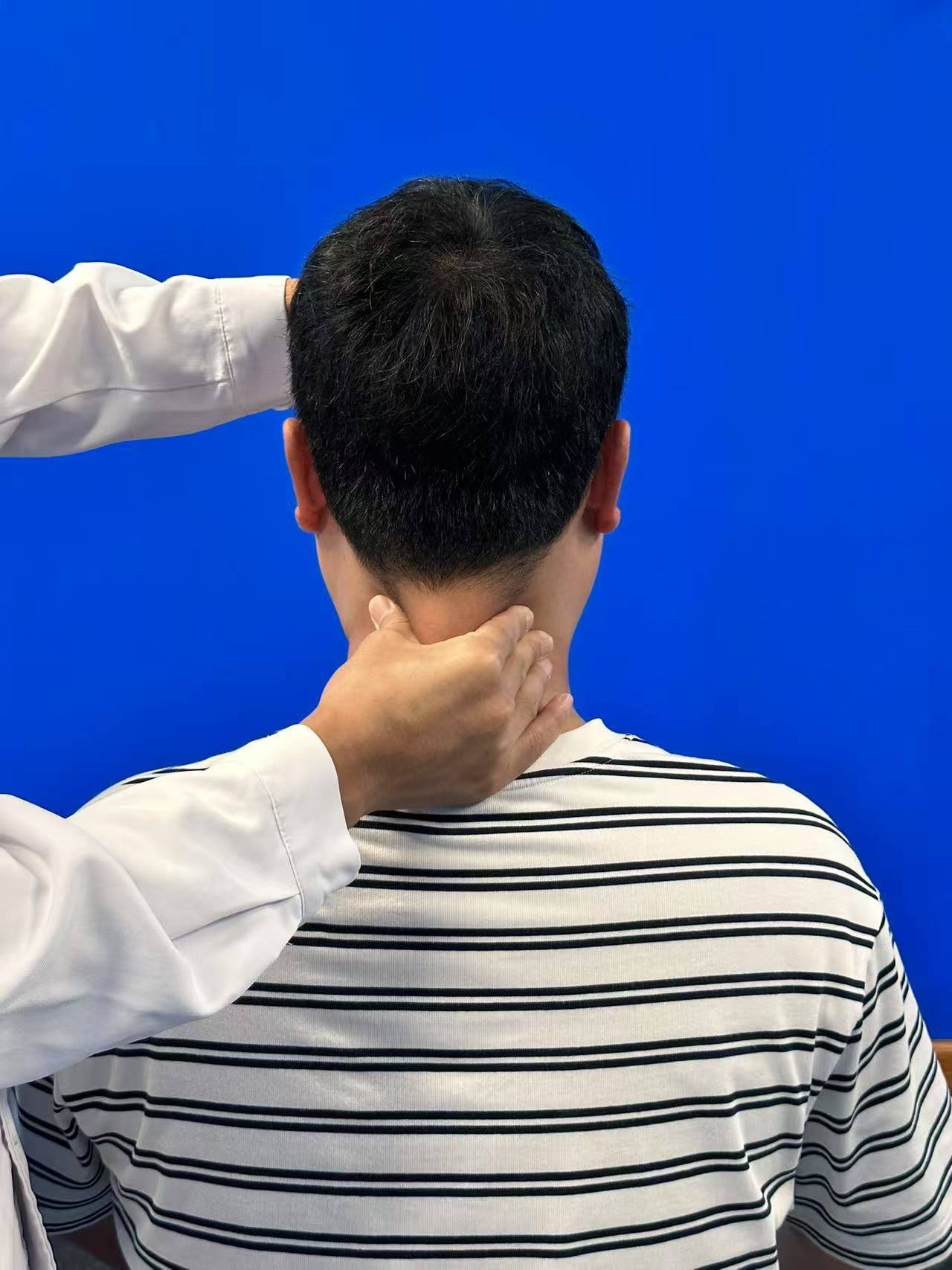  (d) |
| (a) Rolling manipulation**.** Contact is established using the dorsal prominence of the fifth metacarpophalangeal joint. A gentle, continuous rotational pressure is applied to the pre-marked area through active pushing-rotating movements generated by the forearm and wrist. This technique is performed at a frequency of 120 to 160 movements per minute. The procedure duration is 5 minutes.  (b) Pressing-kneading manipulation. This technique combines the pressing and kneading manipulations, utilizing the thumb pad for contact with the treatment area. A gentle and rhythmic pressure is applied to the target region through active circular motions generated by the digit. The recommended frequency ranges from 120 to 160 movements per minute. The procedure duration is 3 minutes. The application area remains consistent with (a).  (c) Plucking manipulation. The thumb tip contacts the participations’ cervical muscle groups, applying unidirectional or reciprocating pushing movements perpendicular to the target area (Trapezius, erector spinae and sternocleidomastoid muscle). The procedure duration is 2 minutes.  (d) Grasping manipulation. Place the thumb and the other 4 fingers on either side of the desired operating area (Neck erector spinae muscle or shoulder trapezius muscle). Generating a lifting-kneading motion through opposing forces applied by the thumb and other fingers. Gradual upward pressure is implemented. The procedure duration is 1 minute. | |

**Step 2. Acupressure manipulation**

| 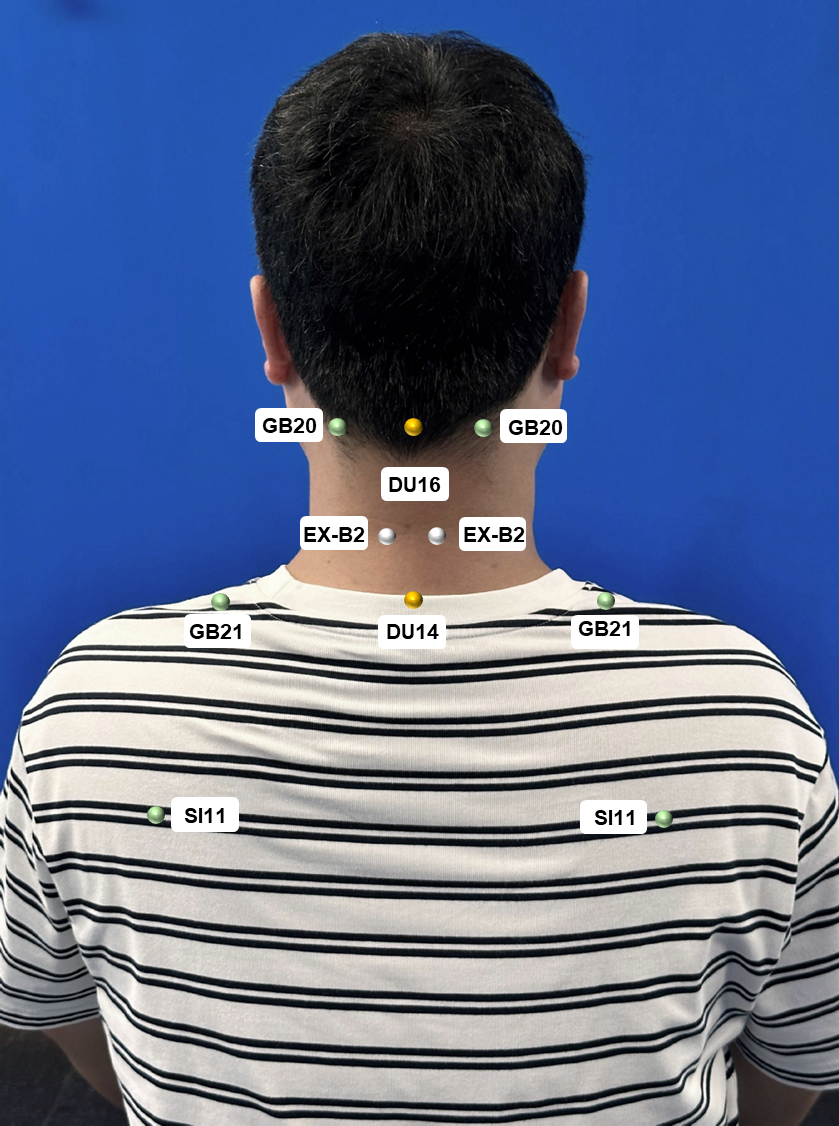  (a) | 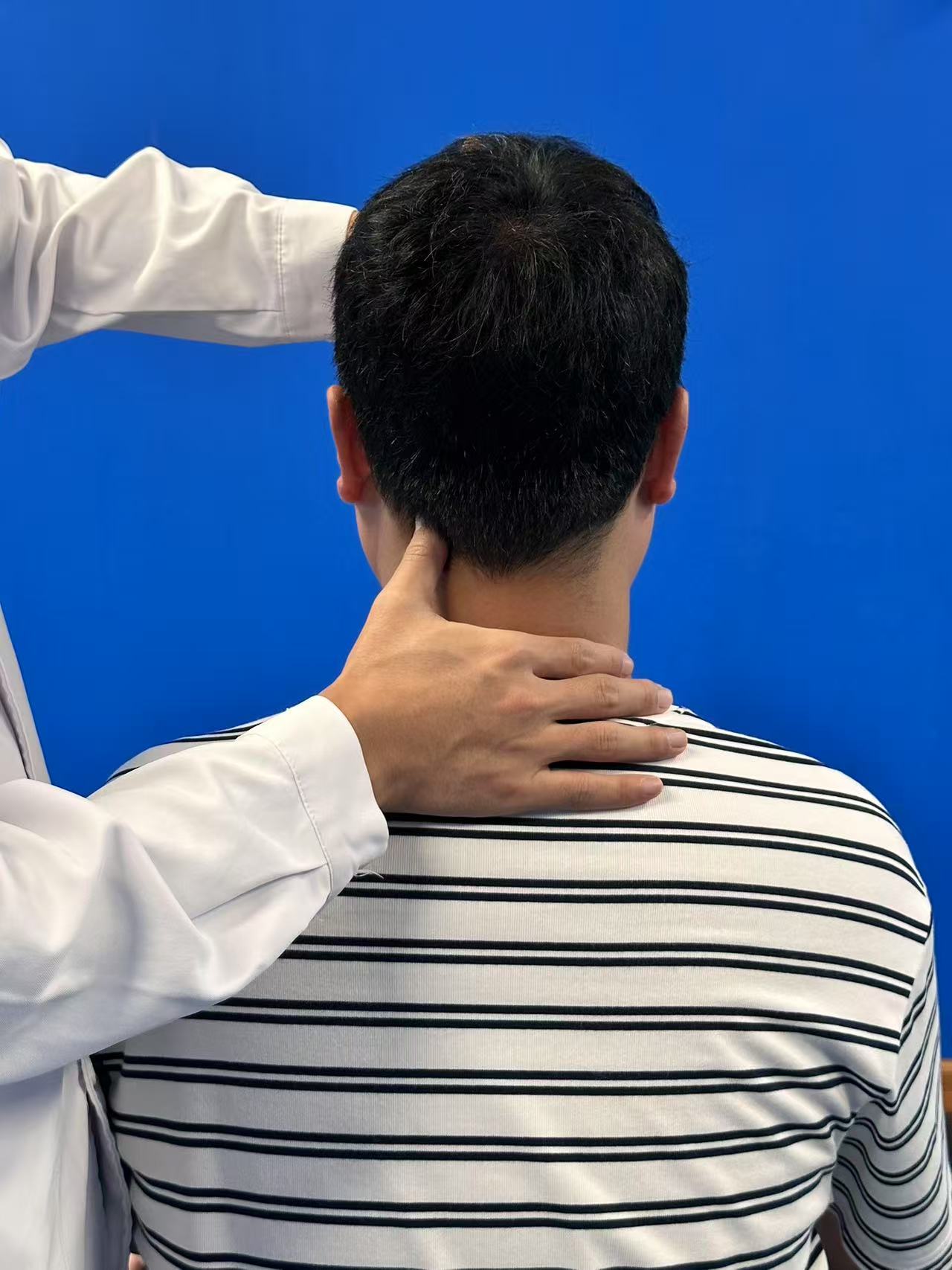  (b) |
| --- | --- |
| (a) Illustration of acupoint locations for Tuina intervention. GB20, DU16, EX-B2, DU14, GB21, SI11.  (b) Pressing manipulation. The thumb pad contacts the skin of patient. Vertical pressure is applied to the acupoint with gradually increasing force until deqi sensation (soreness, numbness, or distension) is elicited. The procedure duration is 1 minute per acupoint. | |

**Step 3. Spinal manipulation**

| **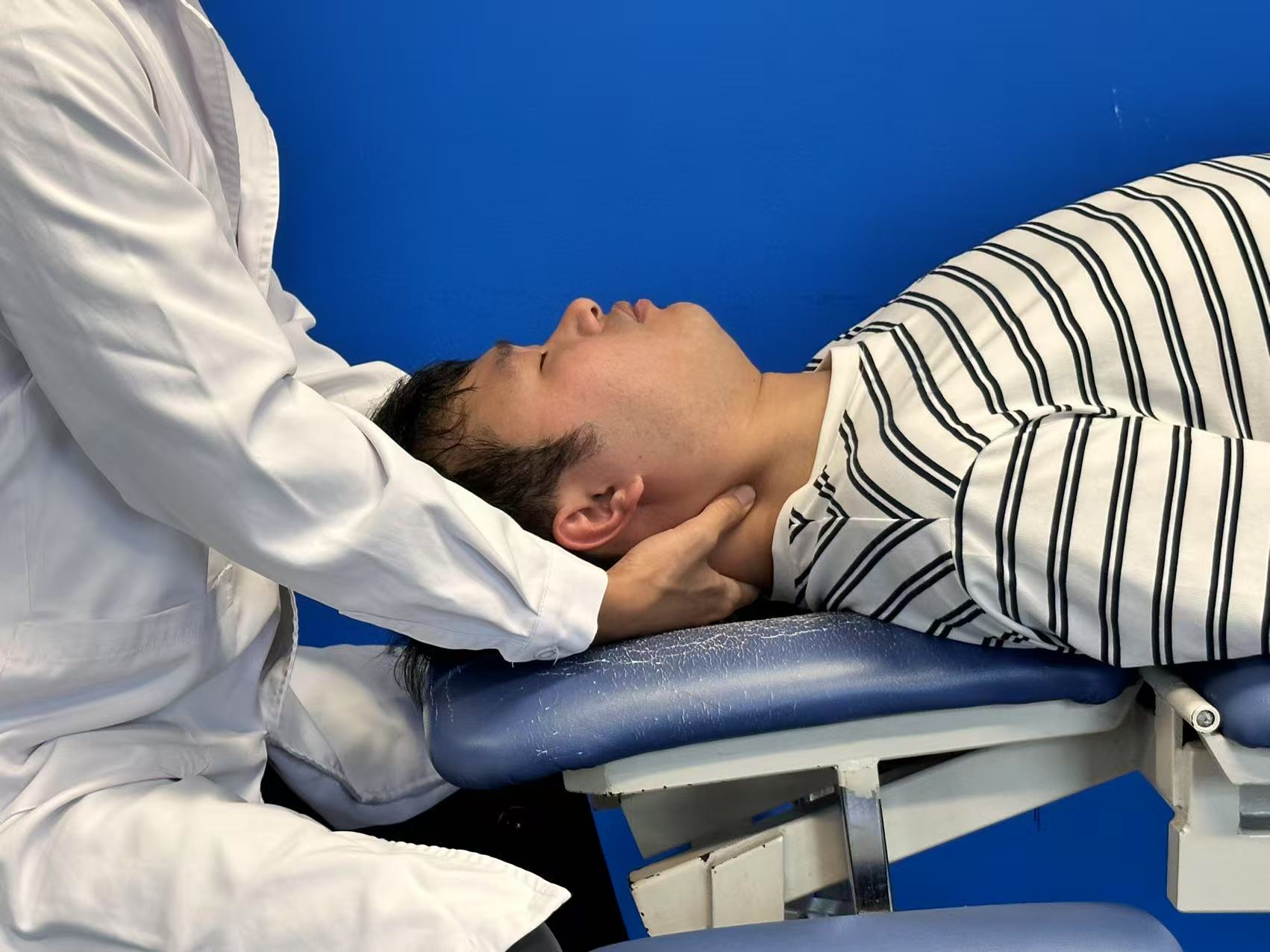**  (a) | **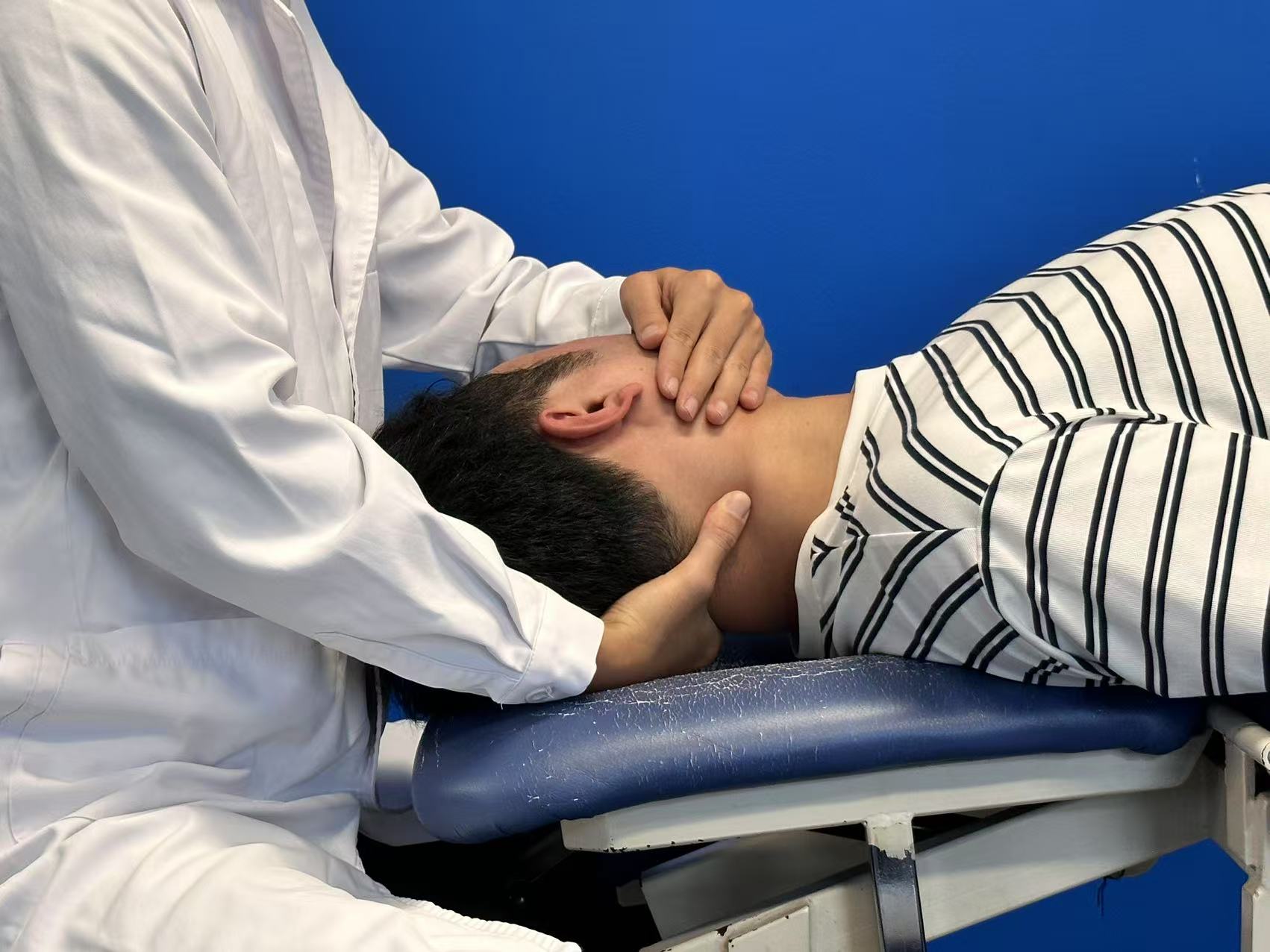**  (b) |
| --- | --- |
| **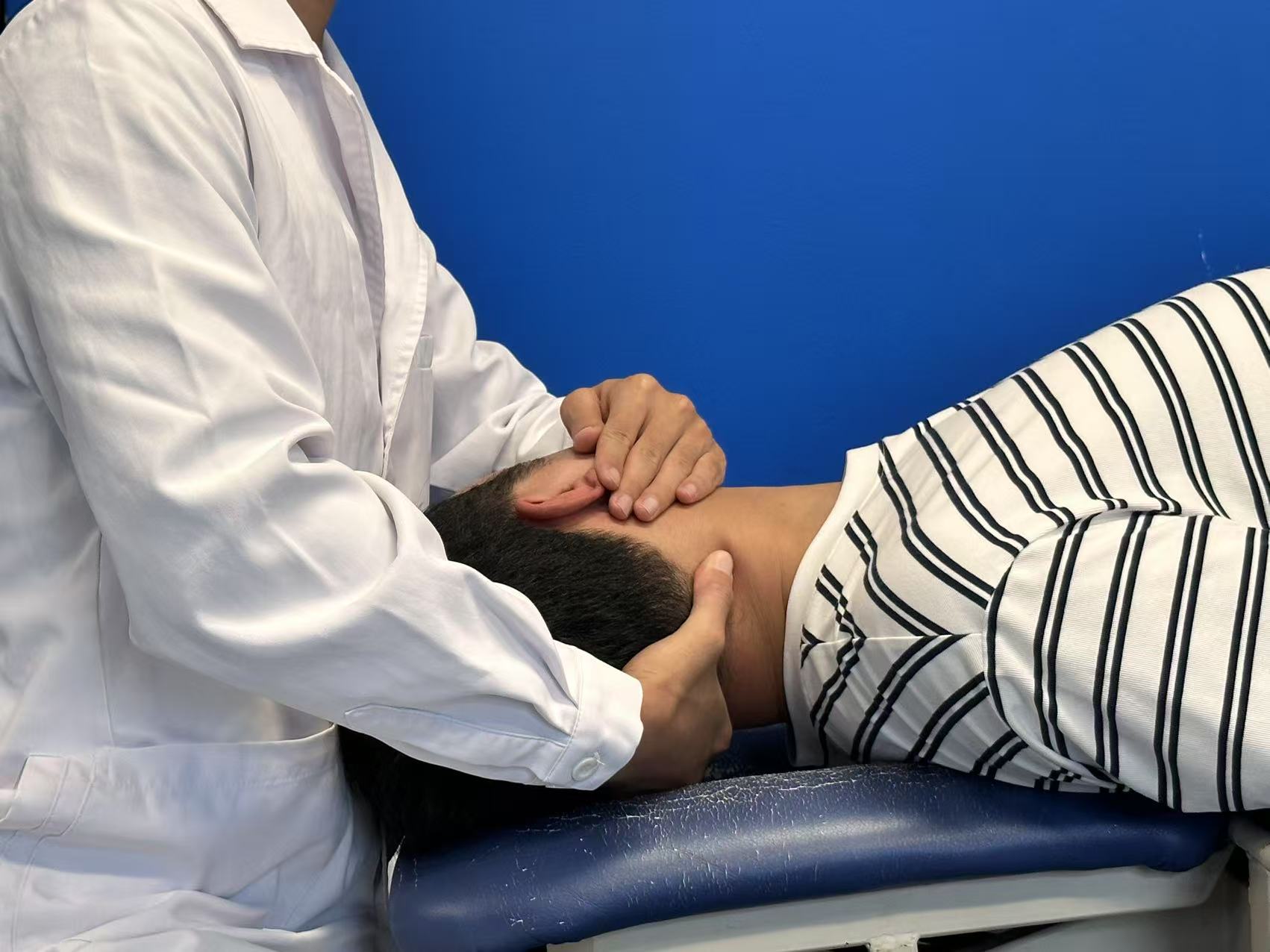**  (c) | **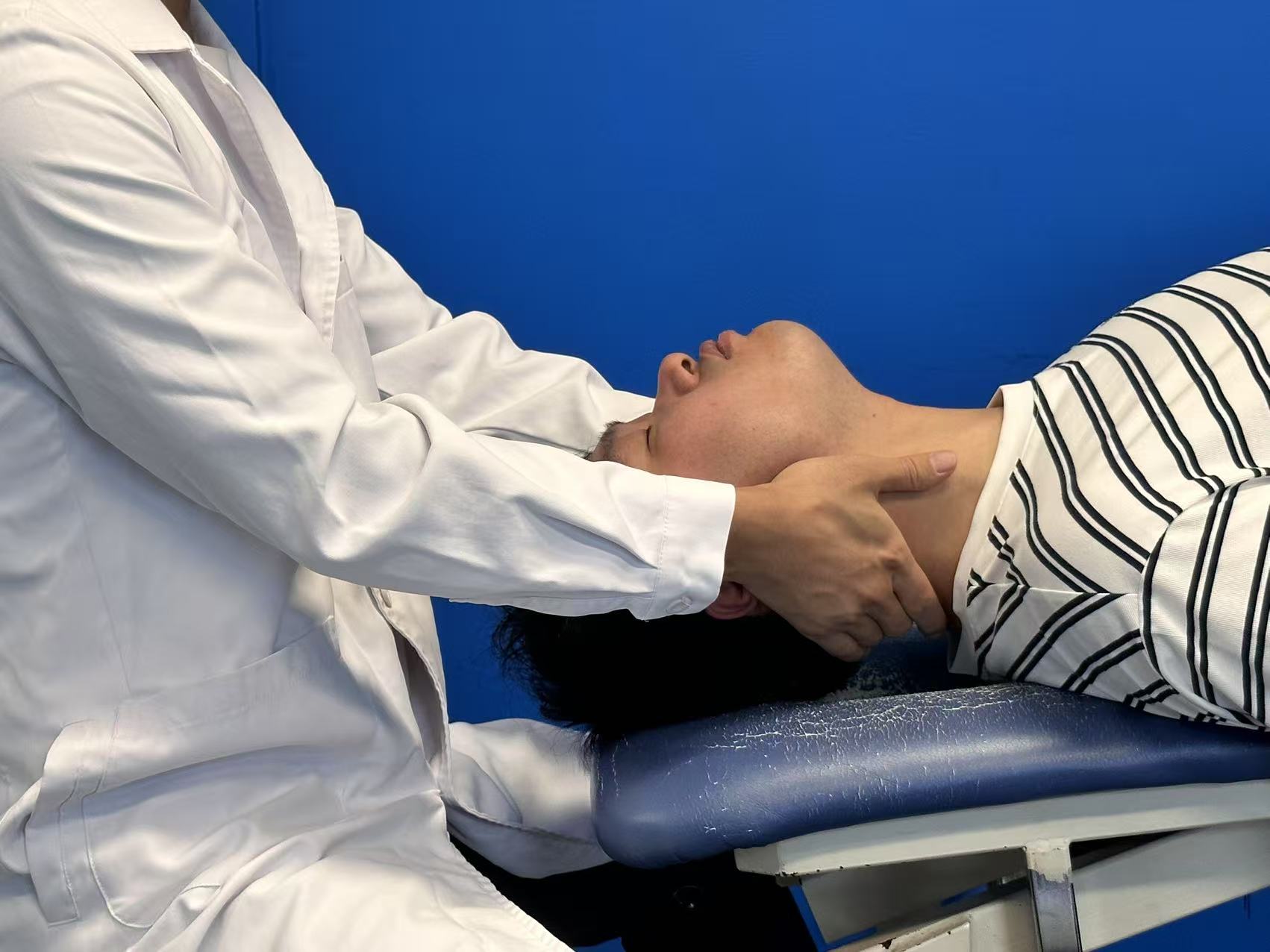**  (d) |
| (a-c) Adjustment of cervical facet joint disorders. The patient lies supine with the neck relaxed and head in neutral position. The therapist sits at the head of the patient, supporting the occiput with one hand while positioning the thumb on the targeted facet joint. The other hand cradles the mandible. The cervical spine is guided into rotation, lateral flexion, and extension to localize mechanical stress to the target segment. A coordinated, low-amplitude, high-velocity rotational thrust is applied, potentially accompanied by an audible click. The procedure duration is 1 minute.  (d) Supine position cervical spine extraction and extension. The patient remains supine with neutral head position. The therapist supports the occiput with both hands and gently elevates the neck to induce slight extension. Synchronized, slow, and horizontal traction is applied along the cervical axis. The procedure duration is 2 minutes. | |
